# Diversity of PBI-DdeI satellite DNA in snakes correlates with rapid independent evolution and different functional roles

**DOI:** 10.1038/s41598-019-51863-w

**Published:** 2019-10-29

**Authors:** Ratchaphol Thongchum, Worapong Singchat, Nararat Laopichienpong, Panupong Tawichasri, Ekaphan Kraichak, Ornjira Prakhongcheep, Siwapech Sillapaprayoon, Narongrit Muangmai, Sudarath Baicharoen, Sunutcha Suntrarachun, Lawan Chanhome, Surin Peyachoknagul, Kornsorn Srikulnath

**Affiliations:** 1 Laboratory of Animal Cytogenetics and Comparative Genomics (ACCG), Department of Genetics, Faculty of Science, Kasetsart University, Bangkok, 10900 Thailand; 2 Interdisciplinary Program in Genetic Engineering, Graduate School, Kasetsart University, Bangkok, 10903 Thailand; 3 Special Research Unit for Wildlife Genomics, Department of Forest Biology, Faculty of Forestry, Kasetsart University, Chatuchak, Bangkok 10900 Thailand; 4 Department of Botany, Faculty of Science, Kasetsart University, Bangkok, 10900 Thailand; 5 Department of Fishery Biology, Faculty of Fisheries, Kasetsart University, Bangkok, 10900 Thailand; 6 Bureau of Conservation and Research, Zoological Park Organization under the Royal Patronage of His Majesty the King, Bangkok, 10300 Thailand; 7 Queen Saovabha Memorial Institute, The Thai Red Cross Society, Bangkok, 10330 Thailand; 8 Center for Advanced Studies in Tropical Natural Resources, National Research University-Kasetsart University, Kasetsart University, (CASTNAR, NRU-KU, Thailand), Bangkok, 10900 Thailand; 9 Center of Excellence on Agricultural Biotechnology (AG-BIO/PERDO-CHE), Bangkok, 10900 Thailand; 10 Omics Center for Agriculture, Bioresources, Food and Health, Kasetsart University (OmiKU), Bangkok, 10900 Thailand

**Keywords:** Cytogenetics, Comparative genomics

## Abstract

To better understand PBI-DdeI satellite DNA located in the centromeric region of python, molecular evolution analysis was conducted on 40 snake species. A ladder-like pattern of DNA bands with repetition of the 194–210 bp monomer was observed in 15 species using PCR. Molecular cloning was performed to obtain 97 AT-rich monomer sequences. Phylogenetic and network analyses showed three PBI-DdeI subfamilies with sequences grouped in species-specific clusters, suggesting rapid evolution. Slow evolution was found in eight species with shared PBI-DdeI sequences, suggesting recent species diversification, allowing PBI-DdeI no time to diverge, with limited homogenization and fixation processes. Quantitative real-time PCR showed large differences in copy number between *Python bivittatus* and other snakes, consistent with repeat scanning of whole genome sequences. Copy numbers were significantly higher in female *Naja kaouthia* than in males, concurring with chromosomal distribution of PBI-DdeI specifically localized to female W chromosomes. PBI-DdeI might act as an evolutionary driver with several repeats to promote W chromosome differentiation and heterochromatinization in *N*. *kaouthia*. Analysis revealed PBI-DdeI with a reduced copy number, compared to *P*. *bivittatus*, in most snakes studied, and it is possible that it subsequently dispersed and amplified on W chromosomes with different functional roles in *N*. *kaouthia*.

## Introduction

Several recent snake genome analyses have revealed that the remarkable variability in genome size results from large differences in the amount of repeated sequences^1^. Tandem repeats make up a large fraction of the genome, with satellite DNA (satDNA) constituting a major part of tandem repeat sequences. The repeats are organized as homogeneous long arrays of head-to-tail orientation, located in the heterochromatic regions of chromosomes such as centromeres and telomeres^2–6^. Repeat sequences are also found abundantly on the sex chromosomes (Y or W), and this is thought to promote sex chromosome differentiation and heterochromatinization^7–9^. Moreover, satDNAs have also been found with epigenetic functions^10^. Multiple copies of the same satDNA family exhibit higher similarity within a species compared to within the same satDNA family of related species. This is a consequence of molecular drive mechanisms known as concerted evolution^3^. This mechanism has evolved via homogenization throughout copies of the satDNA family and fixation in a sexual population group. However, several satDNA families have a species-specific profile (size, nucleotide sequence, copy number, and complexity) derived from satDNA turnover mechanisms such as unequal crossing over and gene conversion; this has led to the emergence of new specific satDNA families/subfamilies^3,6,7^. Different satDNA families/subfamilies may coexist in the genome of a species and can efficiently change the arrangement of DNA sequences in heterochromatin by replacing one dominant satDNA family with another that is less well represented, differing in nucleotide sequences and/or copy numbers in related species, following “the library model”^11^. This consequence varies among satDNA families based on mutation rate, species, chromosome morphology, population size, and reproductive mode^12^. However, this process occurs rapidly among species, resulting in the expansion of new mutations horizontally throughout the genome of distantly related species^6,13^. This aspect might cause reproductive isolation and species radiation^3^. Intriguingly, centromeric satDNA (cen-satDNA) is considered to be a conserved motif comprising several conserved domains such as the CENP box for chromosome stability^14^. By contrast, cen-satDNA is likely to have evolved faster than satDNA of other parts of the chromosomal region^2,15^, leading to the use of phylogenetic markers among diverse lineages.

The investigation of cen-satDNA sequences in squamate reptiles is far from being an in-depth analysis. Such studies have been mostly reported in lacertid and varanid lizards, with a few cases in snakes^4–7,15^. Species-specific variants of satDNA families have been found in lacertid lizards but not in varanid lizards^6,7^. However, evolutionary trends of satDNA within multiple snake species have not yet been well examined, even though snakes form the second largest group of extant reptiles^16^. Karyotype variation is relatively small in snakes, which exhibit conserved ZZ/ZW-type sex chromosomes in most species, the exceptions being *Boa imperator*, *Python bivittatus* and *P*. *regius*^17–20^. Snakes are an excellent model to use to increase understanding of cen-satDNA evolution, including the satDNA library hypothesis. Recently, PBI-DdeI cen-satDNA located on all chromosomes was isolated from the Burmese python (*P*. *bivittatus*) and found to be conserved in only the python lineage, based on the limitation of filter hybridization^5^. Here, we seek to improve our understanding of the PBI-DdeI evolutionary mode in snake lineages comprising 40 snake species, using dot-blot hybridization and PCR based approaches. Genome organization of the repeats was also assessed using a Southern blot hybridization. Various DNA fragments of PBI-DdeI were molecularly cloned from snakes to determine their nucleotide sequences. Quantification of PBI-DdeI was performed on different snake species using quantitative real-time polymerase chain reaction (qPCR) and determined *in silico* for copy number of the repeats on nine snake genome sequences. Chromosomal distribution of the satDNA was subsequently identified in snakes. This allowed us to delineate the evolutionary dynamics of PBI-DdeI and investigate its significance.

## Results

### Isolation and characterization of PBI-DdeI

**Figure 1:**
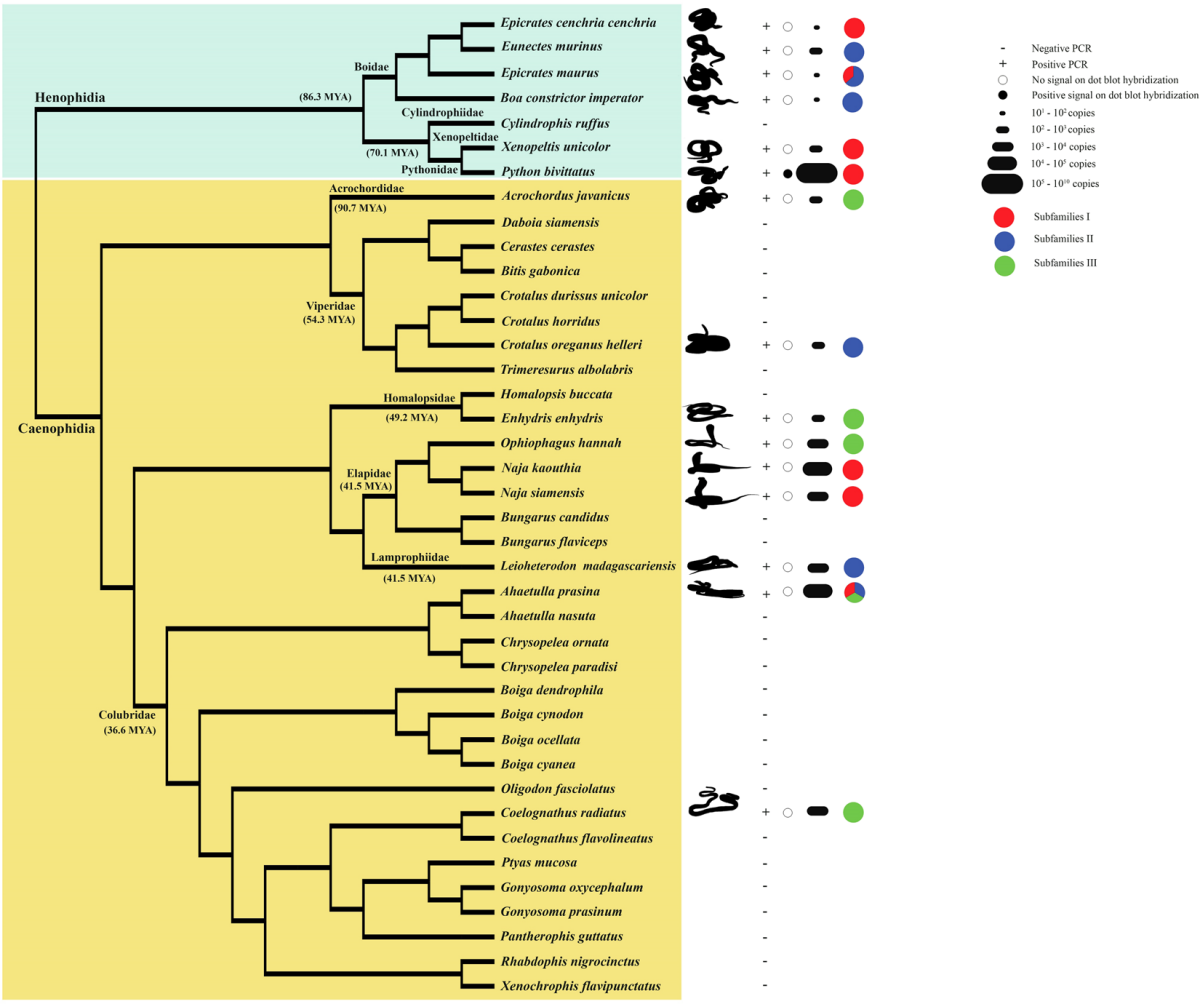
Schematic representation for PBI-DdeI satellite DNA (satDNA) genomic distribution in the snakes studied. Phylogeny was partially derived from Pyron *et al*.^30^. Divergence times were estimated in million years ago for each node^44^. The tree summarizes the results of the dot blot derived from Supplementary Fig. S1, PBI-DdeI copy number is derived from Supplementary Table S7 based on qPCR results, while the classification of satDNA subfamilies is derived from Fig. 2.

Conservation of PBI-DdeI was examined by the dot-blot hybridization of 40 snake species, using their genomic DNA. Intense hybridization signals were only observed for both male and female *P*. *bivittatus* (Supplementary Fig. S1). Specific PBI-DdeI primers were then used to amplify PBI-DdeI sequences in 40 snake species. After gel electrophoresis, PCR products showed a ladder-like pattern of DNA bands, typical of satDNAs, in 15 species (Fig. 1, Supplementary Fig. S2). This pattern was based on the repetition of an approximately 200 bp monomer unit. A total of 97 new sequences of monomer units were obtained with lengths ranging from 194 to 210 bp (Table 1). Several indels (from 12 to 14 bp) were detected (Supplementary Fig. S3). All PBI-DdeI sequences showed AT-bias with an average AT content of 58% and were characterized by possessing a secondary structure (Supplementary Fig. S4). Conserved motifs of PBI-DdeI sequences such as “AACCACGATGTTTTTCTGATTCTACTACCTCG” and “TTTCTGATTCTAC” were found in all sequence units (Supplementary Figs S3 and S5). A BLASTn search of all PBI-DdeI sequences showed similarity, ranging from 70.70% to 90.05%, with PBI-DdeI isolated in a previous study^5^. No significant similarity was found with other sequences deposited in the databases.

**Table 1:** Summary of repeat features and nucleotide diversity, hyplotype number, and hyplotype diversity for each species studied.

Sample	Repeat length (bp)	n	%AT	Nucleotide diversity (π)	Haplotype diversity (*h*)	Accession number
*Epicrates maurus*	196	11	58.70	0.036 ± 0.004	0.945 ± 0.066	LC421903 − LC421913
*Xenopeltis unicolor*	209	5	56.50	0.012 ± 0.005	0.700 ± 0.218	LC421841 − LC421845
*Python bivittatus*	209	4	56.00	0.000 ± 0.000	0.000 ± 0.000	LC421837 − LC421840
*Acrochordus javanicus*	198	8	57.60	0.009 ± 0.002	0.750 ± 0.139	LC421919 − LC421926
*Enhydris enhydris*	196	7	57.10	0.005 ± 0.002	0.476 ± 0.171	LC421927 − LC421933
*Leioheterodon madagascariensis*	194	6	56.50	0.011 ± 0.002	1.000 ± 0.096	LC421875 − LC421880
*Naja kaouthia*	208 − 209	7	56.00	0.019 ± 0.013	0.286 ± 0.196	LC421859 − LC421865
*Naja siamensis*	208 − 209	8	57.90	0.041 ± 0.012	0.750 ± 0.139	LC421851 − LC421858
*Ophiophagus hannah*	208 − 209	8	56.00	0.035 ± 0.006	0.679 ± 0.122	LC421891 − LC421898
*Ahaetulla prasina*	209	9	56.90	0.060 ± 0.006	1.000 ± 0.052	LC421866 − LC421874
*Coelognathus radiatus*	209	7	56.90	0.018 ± 0.004	0.952 ± 0.096	LC421884 − LC421890
*Eunectes murinus*	208 − 210	5	57.90	0.006 ± 0.002	0.600 ± 0.175	LC421846 − LC421850
*Epicrates cenchria cenchria*	209	3	56.00	0.000 ± 0.000	0.000 ± 0.000	LC421881 − LC421883
*Crotalus oreganus helleri*	195	4	57.90	0.003 ± 0.001	0.500 ± 0.265	LC421899 − LC421902
*Boa constrictor imperator*	196	5	58.20	0.004 ± 0.002	0.400 ± 0.237	LC421914 − LC421918

Number of monomeric sequenced repeats (n), nucleotide composition of repeats (AT), length of repeats, nucleotide diversity (π) ± *SD* of each species, haplotype diversity (*h*) ± *SD* of each species, and rate of copy number ± *SD* of each species.

**Figure 2:**
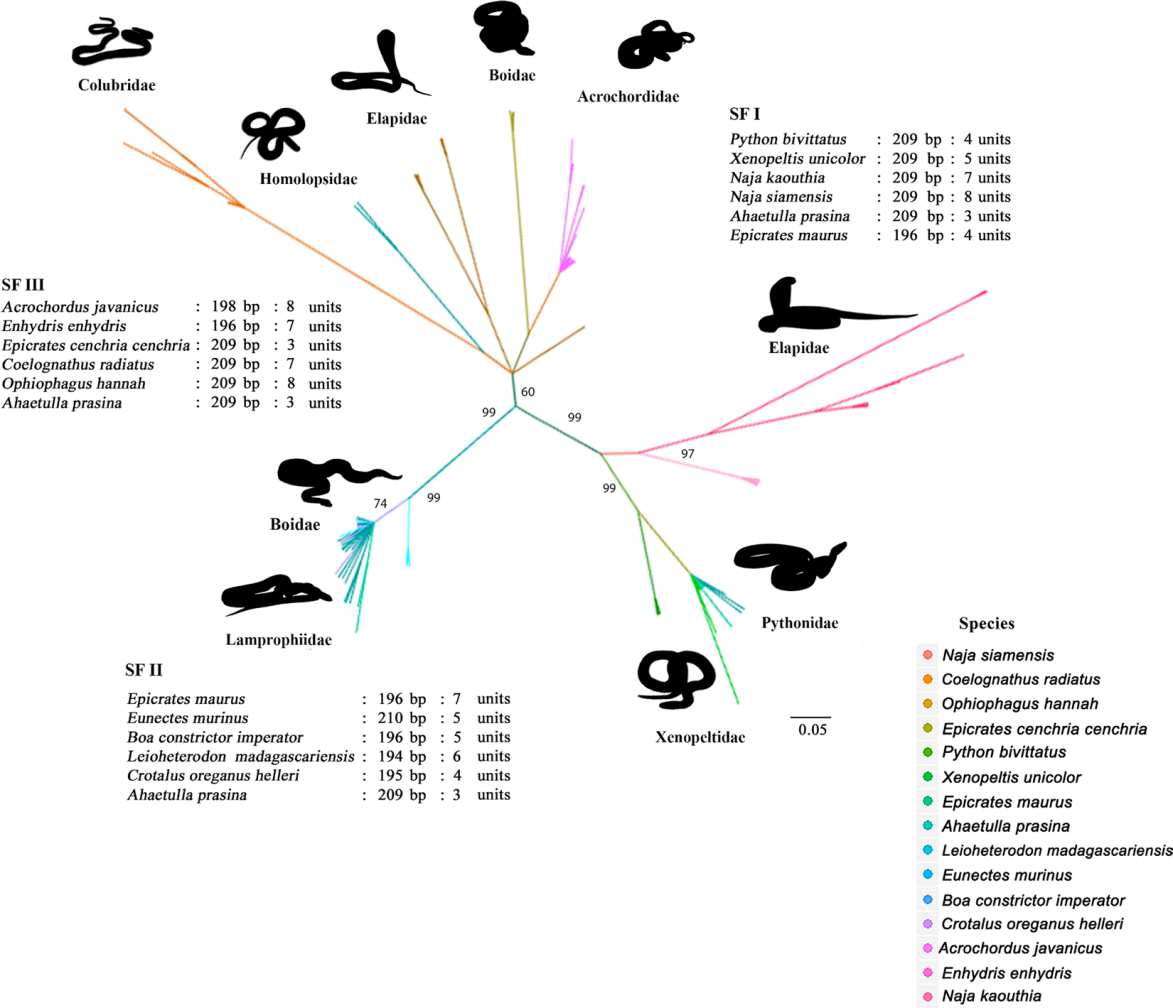
Phylogenetic relationships of PBI-DdeI satellite DNA sequences from fifteen snake species using Bayesian inference analysis. Support values at each node represent Bayesian posterior probability. A colored line indicates different species including subfamilies (subfamily I (SFI), SFII, and SFIII).

A Bayesian unrooted phylogenetic tree was constructed to infer the evolutionary relationship and identify putative PBI-DdeI subfamilies using the PBI-DdeI sequences from all the snakes examined. These subfamilies were defined according to a set of particular nucleotide substitutions or indels. All repeated units were grouped together with three major clades. Subfamily I (SFI) contained 31 sequence units from six snake species. SFII contained 30 units from six snake species. SFIII contained 36 units from six snake species (Fig. 2). Repeated units of *Epicrates maurus* were found in both SFI and SFII, while those of *Ahaetulla prasina* were observed in all subfamilies (Fig. 2, Supplementary Table S1). The evolutionary rate for PBI-DdeI among snake species was about 0.11%/million years (MY) (0.003–0.302%/MY) (Supplementary Table S2). Statistical parsimony network analysis revealed a high level of complex network satDNA sequences within each subfamily. The sequence groups of *P*. *bivittatus*, *N*. *kaouthia* and *N*. *siamensis* tended to be in private specific groups, except for three sequences of *X*. *unicolor*, which were shared with the sequence groups of *E*. *maurus* and *A*. *prasina* in SFI (Supplementary Fig. S6). The sequence groups of *C*. *oreganus helleri*, *B*. *constrictor imperator*, *E*. *murinus*, *E*. *maurus*, and *A*. *prasina* shared a complex network, whereas *L*. *madagascariensis* tended to show the group-specific clade in SFII (Supplementary Fig. S7). Sequence groups of *E*. *cenchria cenchria*, *C*. *radiatus*, *A*. *prasina*, and *O*. *hannah* tended to be in the private specific group, except for *E*. *enhydris*, which overlapped with the sequence groups of *A*. *javanicus* in SFIII (Supplementary Fig. S8).

### Sequence variability of PBI-DdeI within and between species

The average π value was 0.017% ± 0.004 (0.000% ± 0.000 in *P*. *bivittatus* and *E*. *cenchria cenchria*, to 0.060% ± 0.006 in *A*. *prasina*), and the average *h* value was 60.250% ± 13.15 (0.000% ± 0.001 in *P*. *bivittatus* and *E*. *cenchria cenchria*, to 100% ± 0.052 in *A*. *prasina*) (Table 1). The average *p*-distance was 5.850% ± 0.001 (0.009% ± 0.003 between *E*. *enhydris* and *A*. *javanicus*, to 13.200% ± 0.200 between *N*. *siamensis* and *C*. *radiatus*) (Supplementary Table S3). AMOVA analysis of the PBI-DdeI sequences showed 3.090% intra-specific variation (*p* < 0.001) (0.323 of variance components) and 84.678% inter-specific variation (*p* < 0.001) (7.624 of variance components) (Supplementary Table S4).

### Sequence variability of PBI-DdeI within and between subfamilies

The average π value of each PBI-DdeI subfamily was 6.100% ± 0.500 for SFI, 2.600% ± 0.400 for SFII, and 5.700% ± 0.500 for SFIII (Supplementary Table S5), while the average *h* value of SFI was 92.700% ± 2.500, 89.000% ± 5.200 for SFII, and 91.900% ± 3.100 for SFIII. The average *p*-distance between PBI-DdeI subfamilies was 16.200% ± 0.043 for SFI and SFII, 14.200% ± 0.035 for SFI and SFIII, and 10.600% ± 0.033 for SFII and SFIII. AMOVA analysis of PBI-DdeI sequences showed 47.120% molecular variation (*p* < 0.001) (5.450 of variance components) within a subfamily and 22.390% among subfamilies (*p* < 0.001) (2.589 of variance components) (Supplementary Table S6).

### Genomic organization and chromosomal distribution of PBI-DdeI

**Figure 3:**
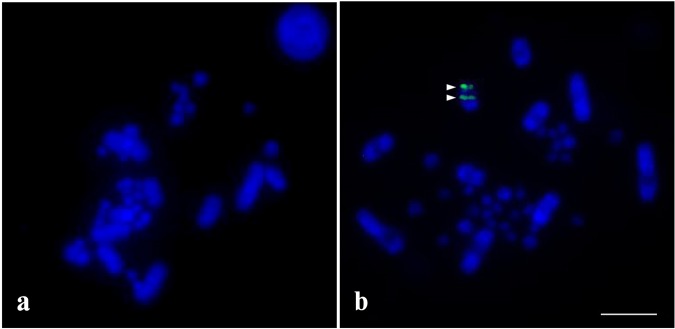
Chromosomal distribution of PBI-DdeI satellite DNA sequences isolated in male (**a**) and female (**b**) *Naja kaouthia*. PBI-DdeI satellite DNA sequences were localized to the W chromosome. Arrows indicate the hybridization signals on the W chromosome. Scale bar represents 10 μm.

Southern blot analyses indicated that PBI-DdeI was organized as tandem arrays in the snake genomes. In the genomic DNA of *P*. *bivittatus* digested with DdeI, hybridization with the PBI-DdeI sequence derived from *P*. *bivittatus*, *N*. *kaouthia*, or *O*. *hannah* produced a polymeric signal ladder based on the 200-bp monomer unit (Supplementary Fig. S9) but this was not found for the genomic DNA of *N*. *kaouthia* and *O*. *hannah* digested with DdeI. For chromosomal localization, the PBI-DdeI sequence was mapped onto female *N*. *kaouthia* chromosomes but not onto male chromosomes (Fig. 3). Strong hybridization signals of PBI-DdeI were localized to the q arm of the W chromosome but not observed on the Z chromosome.

### SatDNA copy number analysis

Copy number quantification of PBI-DdeI showed significant differences between *P*. *bivittatus* and other snake species when examined using both absolute and relative quantification methods (Tukey’s HSD test, *p* ≤ 0.001) (Fig. 1, Supplementary Table S7, Supplementary Fig. S10). Copy number estimates of PBI-DdeI were significantly correlated between absolute and relative quantification methods (Spearman’s *rho* = 0.765, *p* ≤ 0.05) (Supplementary Fig. S11). The nuclear DNA content of female *P*. *bivittatus* was reported in Castoe *et al*.^21^ with a genome size of about 1.435 Gb, and quantification revealed that at least 82.53% (approximately 5.73 × 10^6^ copies per haploid genome) of the *P*. *bivittatus* haploid genome was composed of PBI-DdeI sequences. The amount of PBI-DdeI in the other species was lower than in *P*. *bivittatus* at around 2.07 × 10^5^. No significant correlation was found among π value, monomer size, AT%, and copy number (Supplementary Fig. S11).

The PBI-DdeI copy number differed significantly between sexes in *N*. *kaouthia*, with females showing a copy number about 313 times higher than that of males (Wilcoxon signed-rank test, W = 121, *p* ≤ 0.001) (Supplementary Fig. S12).

### Survey of the whole genome sequence data of snakes

PBI-DdeI sequences were sought in the genome sequences of snakes. For the *N*. *kaouthia* genome, Illumina HiSeq platform sequencing was performed for female genomic DNA with more than 1,250,000,000 reads (373,317 scaffolds). All snake genome sequences were determined for scaffolds (*P*. *bivittatus* (n = 39,112), *T*. *sirtalis* (n = 7,930), *V*. *berus berus* (n = 25,713), *P*. *guttatus* (n = 883,920), *O*. *hannah* (n = 296,399), *C*. *horridus* (n = 186,068), *C*. *mitchellii pyrrhus* (n = 473,380), and *P*. *flavoviridis* (n = 84,502)). PBI-DdeI sequences were mapped to scaffolds with the percentage of identical matches ≥ 80 and e-value ≤ 1e-50 in *P*. *bivittatus* but not for other snakes. PBI-DdeI sequence represented 0.353% of the *P*. *bivittatus* genome.

## Discussion

Sequence domains in cen-satDNA are generally conserved over very long evolutionary periods^3^. A large number of species were examined for cen-satDNA conservation to better understand the biological role of satDNA in diversity and evolution. The PBI-DdeI was AT-rich, as commonly found in squamate reptiles^7,15^, and widely represented in 15 out of 40 snake genomes from different families. Conserved sequence motifs of PBI-DdeI were found in all sequence units with most characterized by a secondary structure. This might be important for chromatin condensation or interaction between protein and DNA^22^, and suggests that PBI-DdeI plays an important role under selective pressure. The copy number of PBI-DdeI in the other 25 species of snake may be too few to be detected by dot-blot and PCR approaches. Alternatively, the loss of PBI-DdeI in several snakes might result from a stochastic effect due to random genetic drift. A satDNA family may be replaced by another satDNA family/subfamily, known as the library model^3,11^. qPCR analysis showed different copy numbers of PBI-DdeI with approximately 5.73 × 10^6^ copies, accounting for 82.53% of the *P*. *bivittatus* genome. By contrast, PBI-DdeIs were identified in scaffolds, accounting for approximately 0.353% (5.070 Mb) of the *P*. *bivittatus* genome, while PBI-DdeI sequences were not identified at all in any of the genome sequences of snakes. These results collectively suggest that these scaffolds are derived from the centromeric region but are still not yet anchored to chromosomes, reflecting the difficulty of sequencing and assembling repeat-rich chromosomal regions^23^. Southern blot analysis with probe snake species of different origin and PCR results also confirmed that PBI-DdeI sequences were arranged with tandem repeat in the snake genome. However, copy numbers differed considerably among species, leading to the disappearance of ladder-like patterns in *N*. *kaouthia* and *O*. *hannah* genomic DNA, as shown in the Southern blot results. Variation of satDNA copy numbers may influence heterochromatin structure and genome variation^2^. Significant differences in copy numbers among fifteen snake species were observed, based on qPCR analysis, the largest difference of approximately 1.83 × 10^6^ times being between *P*. *bivittatus* and the other fourteen species. PBI-DdeI proportion varied among snake species, ranging between 1.01 × 10^−6^% (*E*. *maurus*) – 82.53% (*P*. *bivittatus*), based on the average genome size of snakes. This suggests that the copy number of the satDNA changes very rapidly, despite species being closely related. This might also indicate reflection of PBI-DdeI satDNA neutral stochastic amplification. PBI-DdeI satDNA was acquired in the genome of the common ancestor of snakes, and subsequently amplified independently after the species diverged.

### Do PBI-DdeI sequences suggest a rapid- or slow-evolving element

The evolutionary turnover rate of satDNA is generally either observed at a rapid (found in closely related species) or slow level (represented in several related species)^24,25^. Here, phylogenetic and complex network analyses showed that the sequence group of *A*. *prasina*, *E*. *maurus*, *B*. *constrictor imperator*, *E*. *murinus*, *A*. *javanicus*, *C*. *oreganus helleri*, *E*. *enhydris*, and *X*. *unicolor* shared sequences among different species, especially *A*. *prasina* and *E*. *maurus*, which shared sequences in different repeated subfamilies. High π and *h* values, and low *p*-distances were also found in *A*. *prasina* and *E*. *maurus*. This suggests that the PBI-DdeI of these species shows slow evolutionary rates (0.075%/MY), similar to the rate of satDNA at 0.040% MY found in *Lacerta bilineata*^7^. By contrast, *P*. *bivittatus*, *N*. *kaouthia*, *N*. *siamensis*, *L*. *madagascariensis*, *O*. *hannah*, *E*. *cenchria cenchria*, and *C*. *radiatus* showed species-specific clusters of PBI-DdeI with low π and *h* values. This suggests a state of rapid evolutionary rate (approximately 0.150%/MY), where homogenization and fixation processes effectively occurred after these species split. The low copy number of the original variant might not be homogenized but can generate a new satDNA sequence variant through several processes such as unequal crossing over and/or gene conversion, which is fixed in each species^11^. It is not clear why some satDNA sequences remained conserved for such long evolutionary periods, while others underwent dynamic species-specific nucleotide changes^22^. A similar pattern of satDNA distribution has been observed in bivalve mollusks (*Venerupis* spp.) and characin fish (*Astyanax* spp.), where several species share distinct types of variants and other species show specific and shared variants^26,27^. Diversification of satDNA sequences might be caused by several factors: genomic regions, populations, species, reproductive mode, recombination rates, and copy number^22,26,27^. The disappearance of species-specific PBI-DdeI clusters in several snake lineages could be explained as the result of recent species diversification and rapid speciation, since the PBI-DdeI sequences in each species would not have had time to diverge. It is thought that snakes diverged relatively recently in Squamata, and speciation appears to have occurred in a short period of time^28^. Alternatively, rates of recombination and mutation play an important role in retaining a high degree of intraspecific sequence homogeneity^29^. This suggests that the processes of homogenization and fixation are not very effective in *A*. *prasina*, *E*. *maurus*, *B*. *constrictor imperator*, *E*. *murinus*, *A*. *javanicus*, *C*. *oreganus helleri*, *E*. *enhydris* and *X*. *unicolor*, and that nucleotide mutations have accumulated more rapidly than homogenization rates, resulting in the absence of species-specific sequences. However, this does not rule out the possibility that PBI-DdeI might have several monomer variants. These may be less homologous satDNA sequences left undetected in distant species. We only analyzed a few variants based on the PCR approach described in this study.

Overall, all PBI-DdeIs did not cluster in the same phylogenetic group of snakes, as identified using nuclear or mitochondrial DNA^30,31^. Based on AMOVA analysis, most different sequence groups of all PBI-DdeI subfamilies are shared by distantly related species. The *p*-distances between species within each repeated subfamily are substantially lower than the π values of snake species when looking at repeat units belonging to different repeated subfamilies, suggesting that PBI-DdeI evolved through a “saltatory” processes under the “library hypothesis”^32^, and divergence of repeats probably resulted in a lack of connection to phylogenetic relationships. An alternative explanation relates to horizontal transfer whereby high nucleotide sequence identities between divergent snakes, incongruence between satDNA and snake mitochondrial or nuclear gene phylogenies^30,31^, and a “patchy satDNA distribution” among related snake lineages were observed. This might also suggest a state of horizontal transfer with PBI-DdeI shared between distantly related species^33^. However, no vector such as insects involved in satDNA horizontal transfer has, as yet, been clearly identified. This hypothesis is purely speculative and requires experimental clarification. A large diversity of PBI-DdeI was found in both nucleotide sequences and genomic abundance in snakes in this study. The presence of PBI-DdeI might therefore reflect variable functional constraints under natural selection, with PBI-DdeI having different functions in each snake genome.

### Does diversity of PBI-DdeI correlate with different functional roles

PBI-DdeI sequences are located in the centromeric region of all chromosomes in *P*. *bivittatus*^5^, which might involve chromosome segregation. However, it was cytogenetically demonstrated that PBI-DdeI was amplified in the interstitial sites of W chromosomes in *N*. *kaouthia* in this study. Multiple interstitial satDNA locations on the chromosome might represent remnants of the ancestral centromeric region at chromosome fusions sites, as found in barking deer (*M*. *muntjac*)^33^. Different chromosomal distributions of PBI-DdeI and different karyotypic features between *P*. *bivittatus* and *N*. *kaouthia* species suggest that the W chromosome of *N*. *kaouthia* was probably involved with evolutionary multiple fusions in the snake lineage^5,9^. By contrast, PBI-DdeI was also found in the male genomic DNA, based on qPCR results, indicating that PBI-DdeI sequences were also found in other chromosomes with a very low copy number. Our results clearly show that the copy numbers of most of the PBI-DdeI considered differed significantly between male and female *N*. *kaouthia*, probably due to differential amplification, with females having on average 313 times more copies than males. PBI-DdeI represents 3.81 × 10^−5^% of the *N*. *kaouthia* female genome and 1.22 × 10^−7^% of the *N*. *kaouthia* male genome. An alternative explanation is that differential PBI-DdeI distribution patterns on *N*. *kaouthia* W sex chromosomes result from amplification and dispersion events from the ancestral snake lineage. The heterochromatic W chromosome of *N*. *kaouthia* comprises several microsatellite repeat motifs and telomeric (TTAGGG)_n_ repeats, with BACs containing repeats being amplified on the long arm of the W chromosome^9^. This, in turn, suggests that the W chromosomes of *N*. *kaouthia* have a structurally complex origin containing various repeat sequences on the female-specific region. Co-opted PBI-DdeI acts as an evolutionary driver with several repeats to promote W sex chromosome differentiation and heterochromatinization. Accumulations of such repeats are common features on sex chromosomes and have been reported for the sex chromosomes of many vertebrates^8,9,34^. A high rate of sequence homogenization was also found in PBI-DdeI derived from *P*. *bivittatus* and *N*. *kaouthia*. Both species showed a high proportion of private sequence groups in SFI, indicating the concerted evolution of PBI-DdeI. This might result from the influence of chromosomal location because both centromeres and sex chromosomes exhibit not only low rates of recombination but also show critical functions for chromosome stability, segregation, and sex chromosome differentiation^8,9^. These results lead us to predict that PBI-DdeI located in the centromeric regions of most chromosome pairs in the common ancestor of python^5^ has a lower copy number in many snakes, and is subsequently dispersed and amplified on the W chromosome with different functional roles in *N*. *kaouthia*. Further study is required to elucidate the molecular mechanisms of PBI-DdeI dispersion in snake lineages, with possibilities including extra-chromosomal circular process DNAs or transposable element arrest processes^35,36^.

Our study provides evidence for the existence of PBI-DdeI in snakes shared by distantly related species, implying differential chromosomal location and repeat copy number in satDNA evolution. The large diversity of PBI-DdeI may have several different functions, including a critical role in genome evolution. Further studies of the genome-wide variability and organization of reptilian satDNAs are required to test current hypotheses and identify the mechanisms influencing the evolution of this genomic component. Our results advance our understanding of the organization, diversification, evolution and possible role of satDNA sequences in the genome.

## Materials and Methods

### Specimen collection and DNA extraction

All snake samples, comprising 40 species in total, were collected from the Queen Saovabha Memorial Institute (Bangkok) and Real Zoo (Ayutthaya). Detailed information is presented in Supplementary Table S8. The sex of each individual was identified morphologically and confirmed using a molecular sexing approach^18–20^. Blood samples were collected from the ventral tail vein using a 23-gauge needle attached to 2-ml disposable syringes. These contained either 10 mM ethylenediaminetetraacetic acid (EDTA) for DNA extraction or 75 USP unit/ml heparin for cell culture^9,37^. Whole genomic DNA was extracted following the standard salting-out protocol as described previously by Supikamolseni *et al*.^38^ and used as templates for molecular sexing. Animal care and all experimental procedures were approved by the Animal Experiment Committee, Kasetsart University, Thailand (approval no. ACKU61-SCI-024) and conducted in accordance with the Regulations on Animal Experiments at Kasetsart University.

### Molecular cloning of PBI-DdeI sequence based on PCR strategy

DNA fragments of PBI-DdeI sequences were amplified using specific primers PBI-DdeIF: 5′-GTTGTGAAAGGGCAGTTTTGCC-3′ and PBIDdeIR: 5′-GCTGATGATTCATGTTCTCCCG-3′, which were designed based on a consensus of PBI-DdeI sequences^5^. PCR amplification was performed using 15 μl of 1 × buffer containing 1.5 mM MgCl_2_, 0.2 mM dNTPs, 0.5 μM of primers, 0.5 U of *Taq* polymerase recombinant (Apsalagen Co. Ltd., Bangkok, Thailand), and 25 ng of genomic DNA. PCR conditions were as follows: an initial denaturation at 94 °C for 3 min, followed by 35 cycles of 94 °C for 30 s, 60 °C for 30 s, 72 °C for 1 min 30 s, and a final extension at 72 °C for 5 min. PCR products were visualized by electrophoresis on 1% agarose gel and molecularly cloned using pGEM-T Easy Vector (Promega Corporation, Madison, Wisconsin, USA). The nucleotide sequences of the DNA fragments were determined using the DNA sequencing services of First BASE Laboratories Sdn Bhd (Seri Kembangan, Selangor, Malaysia). The BLASTn programs (http://blast.ncbi.nlm.nih.gov/Blast.cgi) were used to search for nucleotide sequences in the National Center for Biotechnology Information (NCBI) database to confirm the identity of the amplified DNA fragments.

### Sequence analysis

All satDNA sequences were examined for regions that formed characteristic secondary structures using the RNAfold web server (http://rna.tbi.univie.ac.at/cgi-bin/RNAWebSuite/RNAfold.cgi)^39^. Multiple sequence alignment was performed with multiple sequence comparison by log-expectation (MUSCLE) (http://www.ebi.ac.uk/Tools/msa/muscle/)^40^ using default parameters. After the visual inspection of alignments, sequences were identified as repeated units and then deposited in the DNA Data Bank of Japan (DDBJ; http://www.ddbj.nig.ac.jp/index-e.html) (Table 1). Intraspecific nucleotide diversity (π value), haplotype number, and haplotype diversity (*h*) were estimated using DnaSP v. 5^41^, and also performed at the satDNA subfamily level. The numbers of insertions and deletions (indels) were manually checked for each repeated unit of all the snake species. A consensus sequence based on the total alignment of units in each species, or each satDNA subfamily, was constructed using the BioEdit sequence alignment editor version 7.2.5^42^ and by choosing the most frequent nucleotide at each position. The level of sequence divergence between species, or between satDNA subfamilies, was estimated using uncorrected pair-wise distances (*p*-distances) as implemented in the Molecular Evolutionary Genetics Analysis 7 (MEGA7) software^43^. The rate of PBI-DdeI evolution was determined for the species studied according to the divergence times estimated for snakes by Vidal *et al*.^44^. Phylogenetic analysis was then performed using Bayesian inference with MrBayes v3.0b4^45^. The Markov chain Monte Carlo process was used to run four chains simultaneously for one million generations, sampling every 100 generations. Log-likelihood and parameter values were assessed with Tracers ver. 1.5^46^. A burn-in of 25% of saved trees was removed, and the remaining trees were used to generate a majority-rule consensus tree with average branch lengths. The Bayesian posterior probability in the sampled tree population was obtained in percentage terms. A phylogenetic network of the consensus sequences was constructed using statistical parsimony generated in PopART v1.7^47^. Analysis of molecular variance (AMOVA)^47^ was used to detect genetic differentiation among satDNA sequences by determining molecular variance and calculating F-statistics using ARLEQUIN 2.000 with 1,000 permutations^48,49^. This was performed at two hierarchical levels to establish how satDNA sequence variability was distributed both within and between the snake species analyzed, and within and between the satDNA subfamilies detected. Spearman’s correlation tests implemented in the statistical software R Version 3.4.3 with the “stats” package were also performed to calculate Spearman’s rank correlation coefficients for satDNA monomer size, A + T content, copy number, and π value^50^.

### Filter hybridization (dot-blot hybridization and Southern blot hybridization)

Dot-blot hybridization was performed to examine the conservation of PBI-DdeI among 40 different snakes as described previously^6^. To prepare the dot blots, 200 ng of genomic DNA was denatured with 0.4 N NaOH for 10 min and then transferred onto a nylon membrane. DNA fragments of PBI-DdeI sequences derived from *P*. *bivittatus* were labeled with DIG-11-dUTP using PCR DIG Labeling Mix (Roche Diagnostics GmbH, Sandhofer, Mannheim, Germany) and standard universal M13 primers, according to the manufacturer’s instructions, then hybridized to the membranes at 45 °C overnight in DIG Easy Hyb solution (Roche Diagnostics GmbH). After hybridization, the membranes were washed at 45 °C in 0.1% sodium dodecyl sulfate (SDS)/2 × saline-sodium citrate (SSC), 0.1% SDS/1 × SSC, 0.1% SDS/0.5 × SSC, and 0.1% SDS/0.1 × SSC for 15 min each^6^. Chemiluminescent signals were detected using anti-digoxigenin-AP Fab fragments and CDP-Star and exposed to KODAK T-MAT G/RA dental film (Carestream Health, Rochester, NY, USA). For Southern blot hybridization, three snake species (*P*. *bivittatus*, *Naja kaouthia*, and *Ophiophagus hannah*) showing positive results during PCR detection (see results section) were randomly selected to examine the genome organization of PBI-DdeI. Three micrograms of each genomic DNA were digested with endonucleases that had restriction sites within the sequences of each repeat, fractionated by electrophoresis on 1% agarose gel, and transferred onto a nylon membrane. SatDNA sequences derived from *P*. *bivittatus*, *N*. *kaouthia*, and *O*. *hannah* were then labeled with DIG-11-dUTP, using PCR DIG Labeling Mix according to the manufacturer’s protocol. All labeled probes were reciprocally hybridized to membranes of all the three snake species at 45 °C overnight in DIG Easy Hyb solution. Hybridization and detection of signals were performed as mentioned previously.

### Cell culture and chromosome preparation

Lymphocytes from two male and two female *N*. *kaouthia* were isolated from peripheral blood, and then cultured for 5 days in RPMI 1640 medium supplemented with 15% fetal bovine serum (FBS), 3 μg/ml concanavalin A (type IV-S) (Sigma-Aldrich, St. Louis, MO, USA), 10 μg/ml lipopolysaccharide (Sigma-Aldrich), 1% phytohaemagglutinin (HA15) (Remel, Lenexa, KS, USA), and 1% Antibiotic-Antimycotic (Life Technologies-Gibco, Carlsbad, CA, USA) as described previously^9^. After 5 days, lymphocytes were subjected to colcemid treatment (100 ng/ml) for 60 min and fixed (3:1 methanol/acetic acid) after hypotonic treatment in 0.075 M KCl before being harvested. The cell suspension was dropped onto clean glass slides and air-dried. The slides were kept at −80 °C until required for use.

### FISH (fluorescence *in situ* hybridization) mapping of PBI-DdeI

Chromosomal locations of PBI-DdeI were determined using FISH as described previously^9,51,52^. Approximately 250 ng of satDNA fragments were labeled, incorporating biotin-16-dUTP (Roche Diagnostics) by nick translation according to the manufacturer’s protocol, and ethanol-precipitated with salmon sperm DNA and *Escherichia coli* tRNA. After the hybridization of the biotin-labeled probes to *N*. *kaouthia* chromosomes, the probes were detected by incubating the chromosome slides with avidin labeled with fluorescein isothiocyanate (avidin-FITC; Invitrogen, CA, USA). Slides were subsequently stained with 1 µg/ml DAPI (4′, 6′-diamidino-2-phenylindole). Fluorescence hybridization signals were captured using a cooled Charge-Coupled Device (CCD) camera mounted on a ZEISS Axioplan2 microscope and processed using MetaSystems ISIS v.5.2.8 software (MetaSystems, Altlussheim, Germany).

### Quantification of satellite DNA copy number variation based on quantitative real-time polymerase chain reaction (qPCR)

Quantification of PBI-DdeI was performed on different snake species using qPCR with two different approaches: absolute quantification and relative quantification^37,53,54^. PBI-DdeI sequences were amplified using specific primers: PBI-DdeIF and PBI-DdeIR. The glyceraldehyde-3-phosphate dehydrogenase (*GAPDH*) gene was used as the reference with primer sequences GAPDH F (5′-AAACCAGCCAAGTACGATGACAT-3′) and GAPDH R (5′-CCATCAGCAGCAGCCTTCA-3′)^55^. qPCR amplification was performed using 10 μl of 2 × KAPA SYBR® FAST qPCR Master Mix (Kapa Biosystems, Cape Town, South Africa), 0.25 μM primers, and 25 ng of genomic DNA. The PCR conditions were as follows: initial denaturation at 95 °C for 10 min, followed by 40 cycles of 95 °C for 15 s, 60 °C for 15 s, and 72 °C for 15 s, with a final extension at 72 °C for 5 min. A melting curve over a range of temperatures from 60 to 95 °C was created after each run to ensure there was no non-specific product amplification. Amplification specificity was confirmed by dissociation curve analysis. Specificity of the amplified product was additionally tested on 1% agarose gel. No template control was included in any of the runs. Reactions were carried out in a 96-well optical plate and a melt curve was obtained to evaluate primer specificity. The qPCR reactions of all specimens were performed in technical triplicate. For absolute quantification^37^, a 10-fold serial dilution series of the clones (plasmid DNA with the PBI-DdeI clone) ranging from 1 × 10^9^ to 1 × 10^4^ was used to obtain a standard curve (six-point serial dilutions) (Supplementary Fig. S13). Concentration of the recombinant plasmid was obtained using NanoDrop™ 2000/2000c Spectrophotometers (Thermo Fisher Scientific, Massachusetts, USA). The plasmid copy number was calculated using the following equation: DNA (copy number) = [(6.023 × 10^23^) × (Copy number/mol) × DNA amount (g)]/[DNA length (bp) × 660 × (g/mol/bp)]. Avogadro’s number = 6.023 × 10^23^ molecules (copy number/mol) with an average molecular weight of a double-stranded DNA molecule of 660 g/mol/bp. Total DNA length was 3,224 bp [pGEM-T Easy Vector and inserted DNA (PBI-DdeI sequences) were 3,015 and 209 bp]. Cycle threshold (CT) values in each dilution were measured using qPCR to create different standard curves. The standard curve was constructed using cycle threshold values against the log concentration of PBI-DdeI. The regression line was fitted with *R*^2^ = 0.984 (*p* < 0.001). Copy number determination of the unknown total DNA sample was then obtained by interpolating its CT value against the standard curve. Absolute quantification was transformed into fold change values using the standard curve equation and always compared with a reference sample. For relative quantification, the 2^−ΔCT^ method^56^ was used to calculate fold changes in the amount of PBI-DdeI in the different species. Results were represented as the 2^−ΔCT^ of satDNA copy number. Correlation analysis of absolute and relative quantification of PBI-DdeI among snake species using Spearman’s rank correlation coefficient was performed. Copy number differences among snake genome were examined using Analysis of Variance (ANOVA) and the Tukey’s test, using the R statistical software Version 3.4.4 with the “stats” package^50^. Estimated values were expressed as mean ± standard deviation.

To examine copy number differences of PBI-DdeI between males and females, qPCR was performed using ten male and ten female *N*. *kaouthia* samples of genomic DNA, which were selected due to the presence of PBI-DdeI on the W chromosome, as determined by FISH (see results section). PCR condition and quantification were performed as mentioned above. Statistical copy number differences of male and female *N*. *kaouthia* were examined using a Wilcoxon signed-rank test with R statistical software version 3.4.4 with the “stats” package^50^.

### Discovery of PBI-DdeI sequences on the released genome sequence of snakes

Pair-end 100× coverage Illumina HiSeq sequencing was performed for the genomic DNA of one female *N*. *kaouthia*. The sequencing library was prepared by random fragmentation of the DNA sample, followed by 5′ and 3′ adapter ligation. Library fragments sizes were performed using a TruSeq DNA PCR free kit in the range 150 bp. The Illumina HiSeq X Ten generates raw images and base calling through an integrated primary analysis software called RTA 2 (Real Time Analysis 2). The BCL (base calls) binary was converted into FASTQ using Illumina package bcl2fastq2-v2.20.0. The demultiplexing option (–barcode-mismatches) was set to default (value: 1). Low-quality sequences (duplicate reads and adapters) from raw data sequences were removed using a fastq-mc algorithm. High-quality reads were then assembled using Velvet (Velvet_1.1.07; kmer = 91)^57^ with genomic scaffolds used as the database. Whole sequence data was then deposited in a Sequence Read Archive (SRA) (accession number PRJNA506318). The FASTA genome sequences from eight snake species were also retrieved from the NCBI website (http://www.ncbi.nlm.nih.gov) for the Burmese python (*P*. *bivittatus*; AEQU00000000), garter snake (*Thamnophis sirtalis*; LFLD00000000), corn snake (*Pantherophis guttatus*; JTLQ01000000), king cobra (*O*. *hannah*; AZIM00000000), “European adder (*Vipera berus berus*, JTGP00000000)”, timber rattlesnake (*Crotalus horridus*; LVCR00000000.1), speckled rattlesnake (*Crotalus mitchellii pyrrhus*; JPMF01000000), and habu snake (*Protobothrops flavoviridis*; BFFQ00000000.1)^21,58–63^. These genomes were obtained using different sequencing technologies and various levels of characteristics concerning the sequencing coverage and the assembly effort. An in-house computational pipeline, including custom-made BLASTn and sort command, was used to sort and filter the alignments results. Here, PBI-DdeI hits were initially identified in each genome using an iterative query-driven method based on sequence similarity. The consensus sequence of PBI-DdeI was used as the input query following a best-hits filtering algorithm default. This process was repeated three times to accommodate the inclusion of new genomes at various stages in the pipeline. Fragments that had Blast hits with more than 80% (e-value 1 × 10^50^) identity and were longer than 150 bp were calculated as the copy number of PBI-DdeI.

### Accession numbers

LC421837 – LC421933 (DDBJ) and PRJNA506318 (SRA).

## Supplementary information

Supplementary Information
