# Supplementary material for: Diversity of PBI-DdeI satellite DNA in snakes correlates with rapid independent evolution and different functional roles
Source: Sci Rep. 2019 Oct 29;9:15459. doi: 10.1038/s41598-019-51863-w (PMC6820872; doi:10.1038/s41598-019-51863-w)
Supplement: Supplementary file 1 — Supplementary Information [file 41598_2019_51863_MOESM1_ESM.pdf]

Diversity of PBI-DdeI satellite DNA in snakes correlates with rapid independent evolution and different functional roles

Running title: Diversity of satellite DNA in snakes

Ratchaphol Thongchum[1,2,3+], Worapong Singchat[1,3+], Nararat Laopichienpong[1,3], Panupong Tawichasri[1,3], Ekaphan Kraichak[4], Ornjira Prakhongcheep[1], Siwapech Sillapaprayoon[1,3], Narongrit Muangmai[5], Sudarath Baicharoen[6], Sunutcha Suntrarachun[7], Lawan Chanhome[7], Surin Peyachoknagul[1], and Kornsorn Srikulnath[1,3,8,9,10*]

+These authors are joint first authors.

[1]Laboratory of Animal Cytogenetics and Comparative Genomics (ACCG), Department of Genetics, Faculty of Science, Kasetsart University, Bangkok 10900, Thailand

[2]Interdisciplinary Program in Genetic Engineering, Graduate School, Kasetsart University, Bangkok 10903, Thailand

[3]Special Research Unit for Wildlife Genomics, Department of Forest Biology, Faculty of Forestry, Kasetsart University, Chatuchak, Bangkok 10900, Thailand

[4]Department of Botany, Faculty of Science, Kasetsart University, Bangkok 10900, Thailand

[5]Department of Fishery Biology, Faculty of Fisheries, Kasetsart University, Bangkok 10900, Thailand

[6]Bureau of Conservation and Research, Zoological Park Organization under the Royal Patronage of His Majesty the King, Bangkok 10300, Thailand

[7]Queen Saovabha Memorial Institute, The Thai Red Cross Society, Bangkok 10330, Thailand

[8]Center for Advanced Studies in Tropical Natural Resources, National Research University-Kasetsart University, Kasetsart University, Bangkok 10900, Thailand (CASTNAR, NRU-KU, Thailand)

[9]Center of Excellence on Agricultural Biotechnology (AG-BIO/PERDO-CHE), Bangkok 10900, Thailand

[10]Omics Center for Agriculture, Bioresources, Food and Health, Kasetsart University (OmiKU), Bangkok 10900, Thailand

*Corresponding author: kornsorn.s@ku.ac.th

Supplementary Information

Supplementary Table S1 Summary of repeat units and subfamilies in each species.

Supplementary Table S2 Evolutionary rate of PBI-DdeI satellite DNA in the snake species studied.

Supplementary Table S3 Pairwise comparison of PBI-DdeI satellite DNA sequence divergences among fifteen snake species. Species abbreviations were retrieved from Supplementary Table S8.

Supplementary Table S4 AMOVA analysis of PBI-DdeI satellite DNA family within and between species.

Supplementary Table S5 Summary of nucleotide diversity and haplotype diversity in each PBI-DdeI satellite DNA subfamily.

Supplementary Table S6 AMOVA analysis of PBI-DdeI satellite DNA families within and between subfamilies.

Supplementary Table S7 Summary of PBI-DdeI satellite DNA copy number estimates for the snake species studied.

Supplementary Table S8 Summary of the snake species studied.

Supplementary Fig. S1 Dot-blot hybridization patterns of PBI-DdeI satellite DNA. Hybridization membranes probed with PBI-DdeI derived from *Python bivittatus* (a) and representation of their genomic DNA distribution (b). Genomic DNA of 40 snake species were used (Supplementary Table S8). Cloned PBI-DdeIs were used as a control.

Supplementary Fig. S2 Agarose gel electrophoresis of PCR products of PBI-DdeI satellite DNA in five snake species (*Python bivittatus*, *Epicrates maurus*, *Naja siamensis*, *Xenopeltis unicolor*, and *Ahaetulla prasina*). Molecular size of DNA is indicated in the left lane using VC DNA ladder mix (Vivantis Technologies Sdn Bhd, Selangor Darul Ehsan, Malaysia).

Supplementary Fig. S3 Multiple alignment of all PBI-DdeI satellite DNA (satDNA) sequences from the consensus sequences of each species. PBI-DdeI satDNA sequences of fifteen species were used. SF indicates satDNA subfamily. Nucleotide sites occupied by a nucleotide in the consensus sequence are indicated by dots. Nucleotide sites containing different nucleotides are shown as the respective nucleotide observed. The hyphen indicates the absence of a nucleotide at that position.

Supplementary Fig. S4 Secondary structures of PBI-DdeI satellite DNA (satDNA) sequence of fifteen snake species formed using RNAfold web server (http://rna.tbi.univie.ac.at/cgi-bin/RNAWebSuite/RNAfold.cgi). The PBI-DdeI satDNA sequences of fifteen species were

used. SF indicates satDNA subfamily. Species abbreviations were retrieved from Supplementary Table S8.

Supplementary Fig. S5 Multiple alignment of all PBI-DdeI satellite DNA (satDNA) sequences from the consensus sequences of each satDNA subfamily. SF indicates satDNA subfamily. Nucleotide sites occupied by nucleotides in the consensus sequence are indicated by dots. Nucleotide sites containing different nucleotides are shown as the respective nucleotide observed. A hyphen indicates the absence of a nucleotide at that position.

Supplementary Fig. S6 Statistical parsimony network of PBI-DdeI satellite DNA subfamily I, constructed from all sequence units of *Xenopeltis unicolor* (XUN), *Python bivittatus* (PBI), *Naja kaouthia* (NKA), *Naja siamensis* (NSI), *Epicrates maurus* (EMA), and *Ahaetulla prasina* (APR).

Supplementary Fig. S7 Statistical parsimony network of PBI-DdeI satellite DNA subfamily II, constructed from all sequence units of *Epicrates maurus* (EMA), *Eunectes murinus* (EMU), *Ahaetulla prasina* (APR), *Boa constrictor imperator* (BCO), *Crotalus oreganus helleri* (CORE), and *Leioheterodon madagascariensis* (LMA).

Supplementary Fig. S8 Statistical parsimony network of PBI-DdeI satellite DNA subfamily III, constructed from all sequence units of *Coelognathus radiatus* (CRA), *Ophiophagus hannah* (OHA), *Epicrates cenchria cenchria* (ECE), *Acrochordus javanicus* (AJA), *Enhydris enhydris* (EEN), and *Ahaetulla prasina* (APR).

Supplementary Fig. S9 Southern blot hybridization probed with PBI-DdeI satellite DNA sequences derived from *Python bivittatus* (a), *Naja kaouthia* (b) and *Ophiophagus hannah* (c). (a) Hybridization membrane probed with PBI-DdeI satellite DNA sequences derived from *P. bivittatus* to male and female genomic DNA of *P. bivittatus*, *N. kaouthia*, and *O. hannah*. (b) Hybridization membrane probed with PBI-DdeI satellite DNA sequences derived from *N. kaouthia* to male and female genomic DNA of *P. bivittatus*, *N. kaouthia*, and *O. hannah*. (c) Hybridization membrane probed with PBI-DdeI satellite DNA sequences derived from *O. hannah* to male and female genomic DNA of *P. bivittatus*, *N. kaouthia*, and *O. hannah*. M, male F, female

Supplementary Fig. S10 Copy number quantification of PBI-DdeI satellite DNA in fifteen different genomic snake species. Different colored dots and lines indicate different snake species. Error bars represent standard deviations.

Supplementary Fig. S11 Spearman's correlation of AT%, size, copy number (absolute quantification and relative quantification), and intraspecific nucleotide diversity of PBI-DdeI satellite DNA (satDNA) sequence from fifteen snake species. Spearman's correlation tests

were performed among variables. The lower triangular matrix represents scatter plots of the different scores while the upper triangular matrix displays the corresponding Pearson correlation coefficients.

Supplementary Fig. S12 Copy number quantification of PBI-DdeI satellite DNA between male (n = 10) and female (n = 10) genomes of *Naja kaouthia*. Error bars represent standard deviations.

Supplementary Fig. S13 The standard calibration curve used in the absolute quantification of copy number of PBI-DdeI satellite DNA. The linear dynamic range extended from 0.00025 to 25.00 ng/µl of plasmid DNA with the PBI-DdeI clone concentration from *Naja kaouthia* with six different concentrations. Regression lines were calculated as $R^2 = 0.984$, $p < 0.001$.

Supplementary Table S1. Summary of repeat units and subfamilies in each species

Species	Subfamily[1]	Unit
Python bivittatus	SFI	PBI-C3U3, PBI-C4U4, PBI-C1U5, PBI-C2U6
Xenopeltis unicolor	SFI	XUN-C1U1, XUN-C1U2, XUN-C2U3, XUN-C2U4, XUN-C3U5
Naja kaouthia	SFI	NKA-C1U1, NKA-C2U2, NKA-C2U3, NKA-C3U4, NKA-C4U5, NKA-C1U6, NKA-C2U7
Naja siamensis	SFI	NSI-C1U1, NSI-C2U2, NSI-C2U3, NSI-C3U4, NSI-C3U5, NSI-C1U6, NSI-C1U7, NSI-C2U8
Ahaetulla prasina	SFI	APR-C1U1, , APR-C4U4, APR-C4U9
Epicrates maurus	SFI	EMA-C1U2, EMA-C2U4, EMA-C3U6, EMA-C1U9
Eunectes murinus	SFII	EMU-C1U1, EMU-C1U2, EMU-C2U3, EMU-C2U4, EMU-C3U5
Boa constrictor imperator	SFII	BCO-C1U1, BCO-C2U2, BCO-C2U3, BCO-C3U4, BCO-C3U5
Leioheterodon madagascariensis	SFII	LMA-C1U1, LMA-C1U2, LMA-C2U3, LMA-C1U4, LMA-C1U5, LMA-C2U6,
Crotalus oreganus helleri	SFII	CORE-C1U1, CORE-C2U2, CORE-C2U3, CORE-C3U4
Ahaetulla prasina	SFII	APR-C2U2, APR-C3U3, APR-C4U8
Epicrates maurus	SFII	EMA-C1U1, EMA-C2U3, EMA-C3U5, EMA-C4U7, EMA-C1U8, EMA-C2U10, EMA-C3U11
Epicrates cenchria cenchria	SFIII	ECE-C1U1, ECE-C2U2, ECE-C3U3
Ophiophagus hannah	SFIII	OHA-C1U1, OHA-C1U2, OHA-C2U3, OHA-C1U4, OHA-C2U5, OHA-C2U6, OHAC3U7, OHA-C4U8,
Ahaetulla prasina	SFIII	APR-C1U5, APR-C2U6, APR-C3U7
Acrochordus javanicus	SFIII	AJA-C1U1, AJA-C2U2, AJA-C3U3, AJA-C4U4, AJA-C1U5, AJA-C2U6, AJA-C3U7, AJA-C4U8
Enhydris enhydris	SFIII	EEN-C1U1, EEN-C2U2, EEN-C4U4, EEN-C1U5, EEN-C2U6, EEN-C3U7, EEN-C4U8
Coelognathus radiatus	SFIII	CRA-C1U1, CRA-C1U2, CRA-C2U3, CRA-C1U4, CRA-C2U5, CRA-C3U6, CRA-C4U7

[1]SF indicates PBI-DdeI satellite DNA subfamily

Supplementary Tale S2 Evolutionary rate of PBI-DdeI satellite DNA in the snake species studied.

Pair-wise comparison	Average net genetic distance among groups	Divergence in million years	Evolutionary rate
XUN vs PBI	0.040±0.013	70.1	0.056
XUN vs NKA	0.064±0.015	108.7	0.059
XUN vs NSI	0.086±0.017	108.7	0.079
XUN vs APR	0.058±0.011	108.7	0.053
XUN vs EMA	0.089±0.018	96.9	0.092
XUN vs EMU	0.053±0.015	96.9	0.055
XUN vs BCO	0.062±0.016	96.9	0.064
XUN vs CORE	0.061±0.017	108.7	0.056
XUN vs AJA	0.109±0.022	108.7	0.100
XUN vs LMA	0.066±0.017	108.7	0.061
XUN vs CRA	0.124±0.023	108.7	0.114
XUN vs OHA	0.091±0.018	108.7	0.084
XUN vs ECE	0.103±0.022	96.9	0.106
XUN vs EEN	0.106±0.022	108.7	0.097
PBI vs NKA	0.052±0.014	108.7	0.048
PBI vs NSI	0.079±0.017	108.7	0.073
PBI vs APR	0.069±0.013	108.7	0.063
PBI vs EMA	0.107±0.020	96.9	0.111
PBI vs EMU	0.075±0.018	96.9	0.077
PBI vs BCO	0.077±0.018	96.9	0.079
PBI vs CORE	0.076±0.018	108.7	0.070
PBI vs AJA	0.097±0.021	108.7	0.089
PBI vs LMA	0.082±0.019	108.7	0.075
PBI vs CRA	0.118±0.023	108.7	0.108
PBI vs OHA	0.085±0.017	108.7	0.078
PBI vs ECE	0.102±0.022	96.9	0.105
PBI vs EEN	0.094±0.021	108.9	0.086
NKA vs NSI	0.072±0.014	41.5	0.173
NKA vs APR	0.073±0.013	46.3	0.158
NKA vs EMA	0.102±0.019	108.7	0.094
NKA vs EMU	0.075±0.017	108.7	0.069
NKA vs BCO	0.077±0.017	108.7	0.071
NKA vs CORE	0.076±0.017	54.3	0.140
NKA vs AJA	0.079±0.018	90.7	0.087
NKA vs LMA	0.082±0.017	41.5	0.198
NKA vs CRA	0.092±0.019	46.3	0.200
NKA vs OHA	0.072±0.015	41.5	0.174
NKA vs ECE	0.089±0.020	108.7	0.082
NKA vs EEN	0.076±0.018	49.2	0.155
NSI vs APR	0.104±0.016	46.3	0.224
NSI vs EMA	0.142±0.021	108.7	0.131
NSI vs EMU	0.111±0.020	108.7	0.102
NSI vs BCO	0.113±0.020	108.7	0.104
NSI vs CORE	0.112±0.020	54.3	0.207
NSI vs AJA	0.133±0.022	90.7	0.147
NSI vs LMA	0.116±0.020	41.5	0.279
NSI vs CRA	0.132±0.022	46.3	0.286
NSI vs OHA	0.108±0.018	41.5	0.261

Pair-wise comparison	Average net genetic distance among groups	Divergence in million years	Evolutionary rate
NSI vs ECE	0.124±0.023	108.7	0.114
NSI vs EEN	0.130±0.022	49.2	0.264
APR vs EMA	0.095±0.017	108.7	0.087
APR vs EMU	0.048±0.009	108.7	0.044
APR vs BCO	0.050±0.009	108.7	0.046
APR vs CORE	0.049±0.009	54.3	0.090
APR vs AJA	0.105±0.019	90.7	0.116
APR vs LMA	0.055±0.010	46.3	0.118
APR vs CRA	0.111±0.019	36.6	0.302
APR vs OHA	0.080±0.013	46.3	0.174
APR vs ECE	0.090±0.017	108.7	0.083
APR vs EEN	0.102±0.019	49.2	0.208
EMA vs EMU	0.069±0.016	96.9	0.072
EMA vs BCO	0.070±0.016	96.9	0.072
EMA vs CORE	0.069±0.016	108.7	0.063
EMA vs AJA	0.061±0.014	108.7	0.057
EMA vs LMA	0.075±0.016	108.7	0.069
EMA vs CRA	0.146±0.023	108.7	0.135
EMA vs OHA	0.116±0.019	108.7	0.107
EMA vs ECE	0.127±0.022	96.9	0.131
EMA vs EEN	0.058±0.014	108.7	0.054
EMU vs BCO	0.009±0.005	96.9	0.009
EMU vs CORE	0.008±0.005	108.7	0.007
EMU vs AJA	0.097±0.021	108.7	0.089
EMU vs LMA	0.014±0.006	108.7	0.012
EMU vs CRA	0.105±0.021	108.7	0.097
EMU vs OHA	0.074±0.016	108.7	0.068
EMU vs ECE	0.080±0.019	96.9	0.083
EMU vs EEN	0.094±0.021	108.7	0.086
BCO vs CORE	0.003±0.002	108.7	0.003
BCO vs AJA	0.099±0.021	108.7	0.091
BCO vs LMA	0.009±0.004	108.7	0.009
BCO vs CRA	0.108±0.021	108.7	0.099
BCO vs OHA	0.076±0.016	108.7	0.070
BCO vs ECE	0.082±0.019	96.9	0.085
BCO vs EEN	0.096±0.021	108.7	0.088
CORE vs AJA	0.098±0.021	90.7	0.108
CORE vs LMA	0.008±0.004	54.3	0.016
CORE vs CRA	0.107±0.021	54.3	0.197
CORE vs OHA	0.075±0.016	54.3	0.138
CORE vs ECE	0.082±0.019	108.7	0.075
CORE vs EEN	0.095±0.021	54.3	0.176
AJA vs LMA	0.104±0.021	90.7	0.115
AJA vs CRA	0.129±0.024	90.7	0.142
AJA vs OHA	0.097±0.019	90.7	0.107
AJA vs ECE	0.108±0.022	108.7	0.099
AJA vs EEN	0.009±0.003	90.7	0.010
LMAvs CRA	0.113±0.021	46.3	0.243
LMA vs OHA	0.081±0.016	46.3	0.176

Pair-wise comparison	Average net genetic distance among groups	Divergence in million years	Evolutionary rate
LMA vs ECE	0.087±0.019	108.7	0.080
LMA vs EEN	0.101±0.022	49.2	0.205
CRA vs OHA	0.102±0.019	46.3	0.219
CRA vs ECE	0.112±0.022	108.7	0.103
CRA vs EEN	0.125±0.023	49.2	0.253
OHA vs ECE	0.085±0.018	108.7	0.078
OHA vs EEN	0.094±0.019	49.2	0.191
ECE vs EEN	0.105±0.022	108.7	0.096

Supplementary Table S3. Pairwise comparison of PBI-DdeI satellite DNA sequence divergences among fifteen snake species. Species abbreviations were retrieved from Supplementary Table S8.

	XUN	PBI	NKA	NSI	APR	EMA	EMU	BCI	CORH	AJA	LMA	CRA	OHA	ECE	EEN
XUN															
PBI	0.040±0.013														
NKA	0.064±0.015	0.052±0.014													
NSI	0.086±0.017	0.079±0.017	0.072±0.014												
APR	0.058±0.011	0.069±0.013	0.073±0.013	0.104±0.016											
EMA	0.089±0.018	0.107±0.020	0.102±0.019	0.142±0.021	0.095±0.017										
EMU	0.053±0.015	0.075±0.018	0.075±0.017	0.111±0.020	0.048±0.009	0.069±0.016									
BCI	0.062±0.016	0.077±0.018	0.077±0.017	0.113±0.020	0.050±0.009	0.070±0.016	0.009±0.005								
CORH	0.061±0.017	0.076±0.018	0.076±0.017	0.112±0.020	0.049±0.009	0.069±0.016	0.008±0.005	0.003±0.002							
AJA	0.109±0.022	0.097±0.021	0.079±0.018	0.133±0.022	0.105±0.019	0.061±0.014	0.097±0.021	0.099±0.021	0.098±0.021						
LMA	0.066±0.017	0.082±0.019	0.082±0.017	0.116±0.020	0.055±0.010	0.075±0.016	0.014±0.006	0.009±0.004	0.008±0.004	0.104±0.021					
CRA	0.124±0.023	0.118±0.023	0.092±0.019	0.132±0.022	0.111±0.019	0.146±0.023	0.105±0.021	0.108±0.021	0.107±0.021	0.129±0.024	0.113±0.021				
OHA	0.091±0.018	0.085±0.017	0.072±0.015	0.108±0.018	0.080±0.013	0.116±0.019	0.074±0.016	0.076±0.016	0.075±0.016	0.097±0.019	0.081±0.016	0.102±0.019			
ECE	0.103±0.022	0.102±0.022	0.089±0.020	0.124±0.023	0.090±0.017	0.127±0.022	0.080±0.019	0.082±0.019	0.082±0.019	0.108±0.022	0.087±0.019	0.112±0.022	0.085±0.018		
EEN	0.106±0.022	0.094±0.021	0.076±0.018	0.130±0.022	0.102±0.019	0.058±0.014	0.094±0.021	0.096±0.021	0.095±0.021	0.009±0.003	0.101±0.022	0.125±0.023	0.094±0.019	0.105±0.022	

Supplementary Table S4. AMOVA analysis of PBI-DdeI satellite DNA family within and between species

Variation source	d.f.	Sum of squares	Variance components	Percentage variation	F_{st}
Among groups	14	842.691	7.624	72.960	0.729***
Among populations within groups	35	84.678	0.323	3.090	0.114***
Within populations	64	201.500	3.148	30.130	0.698***

*** $p < 0.001$

Supplementary Table S5. Summary of nucleotide diversity and haplotype diversity in each PBI-DdeI satellite DNA subfamily

Subfamily	n	Nucleotide diversity (π)	Haplotype diversity (*h*)	Mean distance within group
I	31	0.061 ± 0.005	0.927 ± 0.025	0.082 ± 0.011
II	30	0.026 ± 0.004	0.890 ± 0.052	0.051 ± 0.008
III	36	0.057 ± 0.005	0.919 ± 0.031	0.085 ± 0.010

Number of monomeric sequenced repeats (n), nucleotide diversity (π) ± *SD* of each repeated subfamily, haplotype diversity (*h*) ± *SD* of each repeated subfamily and mean distance within each group ± *SD* of repeated subfamily

Supplementary Table S6. AMOVA analysis of PBI-DdeI satellite DNA family within and between subfamilies

Variation source	d.f.	Sum of squares	Variance components	Percentage variation	F_{st}
Among groups	2	250.075	2.589	22.390	0.607***
Among populations within groups	12	459.642	5.450	47.120	0.695***
Within populations	83	292.752	3.527	30.490	0.223***

*** $p < 0.001$

Supplementary Table S7. Summary of PBI-DdeI satellite DNA copy number estimates for the snake species studied

Species	n[1]	Unit length (bp)	Relative copy number (copy)	Absolute copy number (copy)	% Genomic proportion
Epicrates maurus	11	196	1.93E+01	8.30E-02	1.20E-06
Xenopeltis unicolor	5	209	4.25E+02	2.80E-01	4.04E-06
Python bivittatus	4	209	3.99E+09	5.73E+06	8.25E+01
Acrochordus javanicus	8	198	4.28E+02	5.98E-01	8.61E-06
Enhydris enhydris	7	196	4.21E+02	9.67E-02	1.39E-06
Leioheterodon madagascariensis	6	194	2.80E+03	7.77E-01	1.12E-05
Naja kaouthia	7	208 – 209	3.52E+04	1.11E+00	1.61E-05
Naja siamensis	8	208 – 209	2.41E+03	1.75E+00	2.52E-05
Ophiophagus hannah	8	208 – 209	6.34E+03	2.77E+01	3.99E-04
Ahaetulla prasina	9	209	7.92E+04	4.28E+00	6.16E-05
Coelognathus radiatus	7	209	4.44E+03	1.90E+00	2.74E-05
Eunectes murinus	5	208 – 210	4.66E+02	8.07E-01	1.16E-05
Epicrates cenchria cenchria	3	209	9.95E+01	8.32E-01	1.20E-05
Crotalus oreganus helleri	4	195	4.34E+02	2.68E+00	3.86E-05
Boa constrictor imperator	5	196	3.66E+01	8.02E-01	1.16E-05

[1]Number of monomeric repeats sequenced (n)

Supplementary Table S8. Summary of snake species studied

Family	Species	Snake group	Abbreviation	Number of animal used (female + male)
Cylindrophiidae	*Cylindrophis ruffus*	Henophidia	CRU	1 + 1
Boidae	*Epicrates maurus*	Henophidia	EMA	0 + 1
	Eunectes murinus	Henophidia	EMU	0 + 1
	Epicrates cenchria cenchria	Henophidia	ECE	0 + 1
	Boa constrictor imperator	Henophidia	BCO	1 + 0
Xenopeltidae	*Xenopeltis unicolor*	Henophidia	XUN	1 + 1
Pythonidae	*Python bivittatus*	Henophidia	PBI	1 + 1
Acrochordidae	*Acrochordus javanicus*	Caenophidia	AJA	1 + 1
Viperidae	*Daboia siamensis*	Caenophidia	DSI	1 + 1
	Cerastes cerastes	Caenophidia	CCE	1 + 1
	Trimeresurus albolabris	Caenophidia	TAL	0 + 1
	Crotalus oreganus helleri	Caenophidia	CORE	0 + 1
	Crotalus durissus unicolor	Caenophidia	CDU	1 + 0
	Crotalus horridus	Caenophidia	CHO	0 + 1
	Bitis gabonica	Caenophidia	BGA	1 + 0
Homalopsidae	*Homalopsis buccata*	Caenophidia	HBU	1 + 1
	Enhydris enhydris	Caenophidia	EEN	1 + 1
Lamprophiidae	*Leioheterodon madagascariensis*	Caenophidia	LMA	1 + 1
Elapidae	*Naja kaouthia*	Caenophidia	NKA	10 + 10
	Naja siamensis	Caenophidia	NSI	1 + 1
	Ophiophagus hannah	Caenophidia	OHA	1 + 1
	Bungarus candidus	Caenophidia	BCA	1 + 1
	Bungarus flaviceps	Caenophidia	BFL	1 + 1
Colubridae	*Oligodon fasciolatus*	Caenophidia	OFA	1 + 1
	Ahaetulla prasina	Caenophidia	APR	1 + 1
	Boiga dendrophila	Caenophidia	BDE	1 + 1
	Gonyosoma oxycephalum	Caenophidia	GOX	1 + 1
	Coelognathus flavolineatus	Caenophidia	CFL	1 + 1
	Xenochrophis flavipunctatus	Caenophidia	XFL	1 + 1
	Coelognathus radiatus	Caenophidia	CRA	1 + 1
	Ptyas mucosa	Caenophidia	PMU	1 + 1
	Pantherophis guttatus	Caenophidia	PGU	1 + 1
	Boiga ocellata	Caenophidia	BOC	0 + 1
	Boiga cyanea	Caenophidia	BCYA	0 + 1
	Boiga cynodon	Caenophidia	BCY	0 + 1
	Rhabdophis nigrocinctus	Caenophidia	RNI	0 + 1
	Ahaetulla nasuta	Caenophidia	ANA	1 + 0
	Chrysopelea paradisi	Caenophidia	CPA	1 + 0
	Chrysopelea ornata	Caenophidia	COR	1 + 0
	Gonyosoma prasinum	Caenophidia	GPR	1 + 0

a

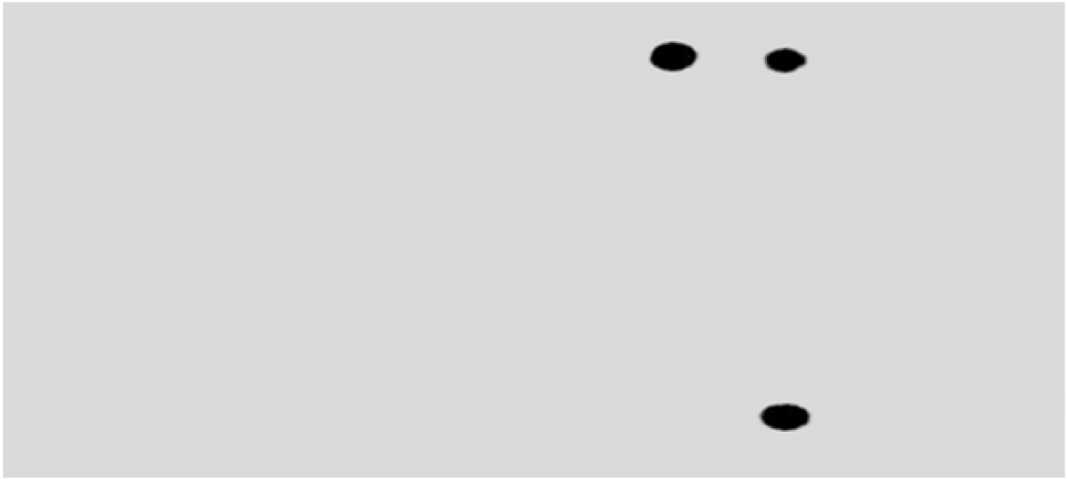

b

CRU-M	CRU-F	EMA-M	EMA-F	XUN-M	XUN-F	PBI-M	PBI-F	AJA-M	AJA-F
DSI-M	DSI-F	CCE-M	CCE-F	HBU-M	HBU-F	EEN-M	EEN-F	LMA-M	LMA-F
PGU-M	PGU-F	NKA-M	NKA-F	NSI-M	NSI-F	OHA-M	OHA-F	BCA-M	BCA-F
BFL-M	BFL-F	OFA-M	OFA-F	APR-M	APR-F	BDE-M	BDE-F	GOX-M	GOX-F
CFL-M	CFL-F	XRA-M	XRA-F	CRA-M	CRA-F	PMU-M	PMU-F	EMU-M	ECE-M
BCO-F	TAL-M	CORE-M	CDU-F	BGA-F	CHO-M	BCY-M	BCO-M	BCYA-M	RNI-M
ANA-F	CPA-F	COR-F	GPR-F				PBI-DdeI satDNA		

Supplementary Figure 1S (Thongchum et al.)

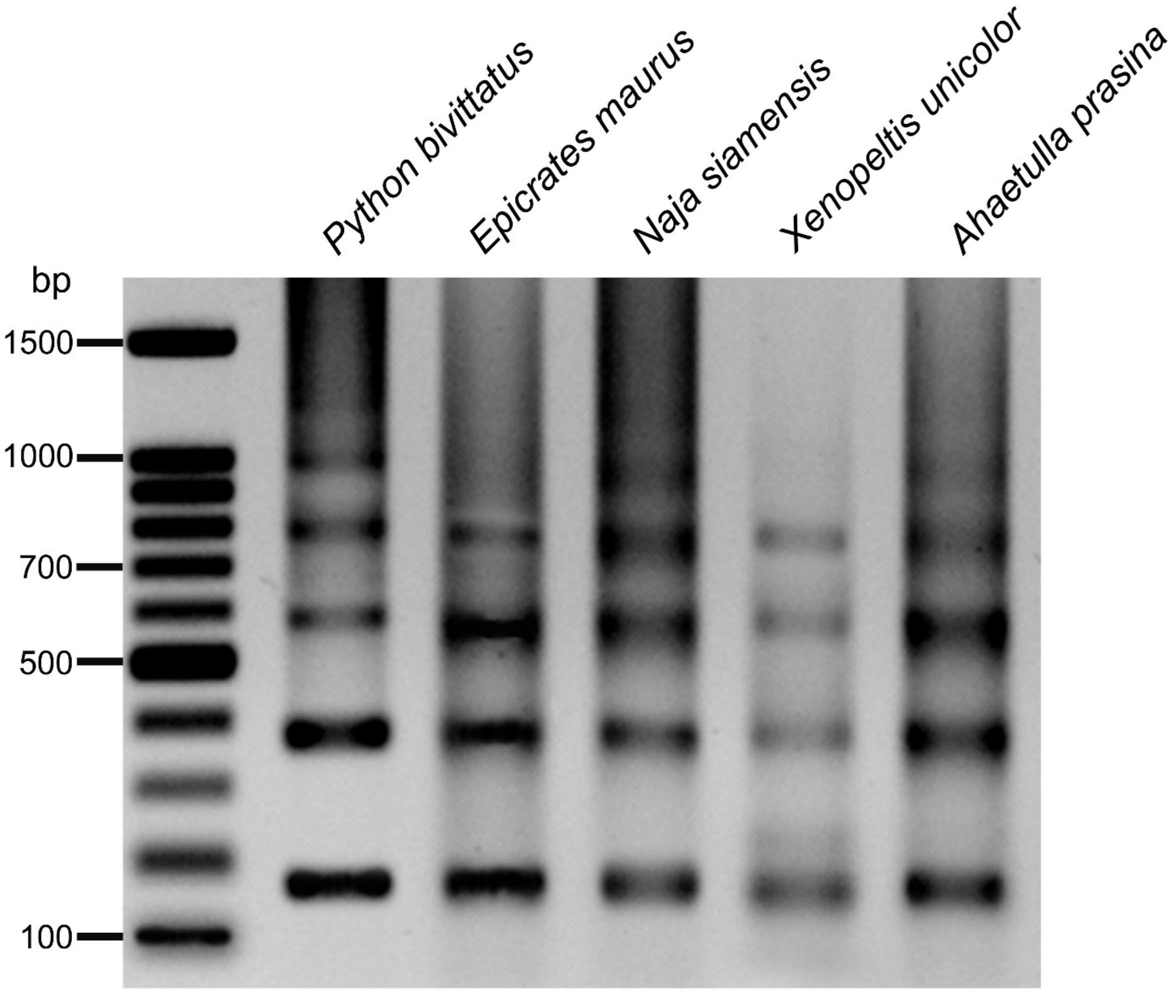

Supplementary Figure 2S (Thongchum et al.)

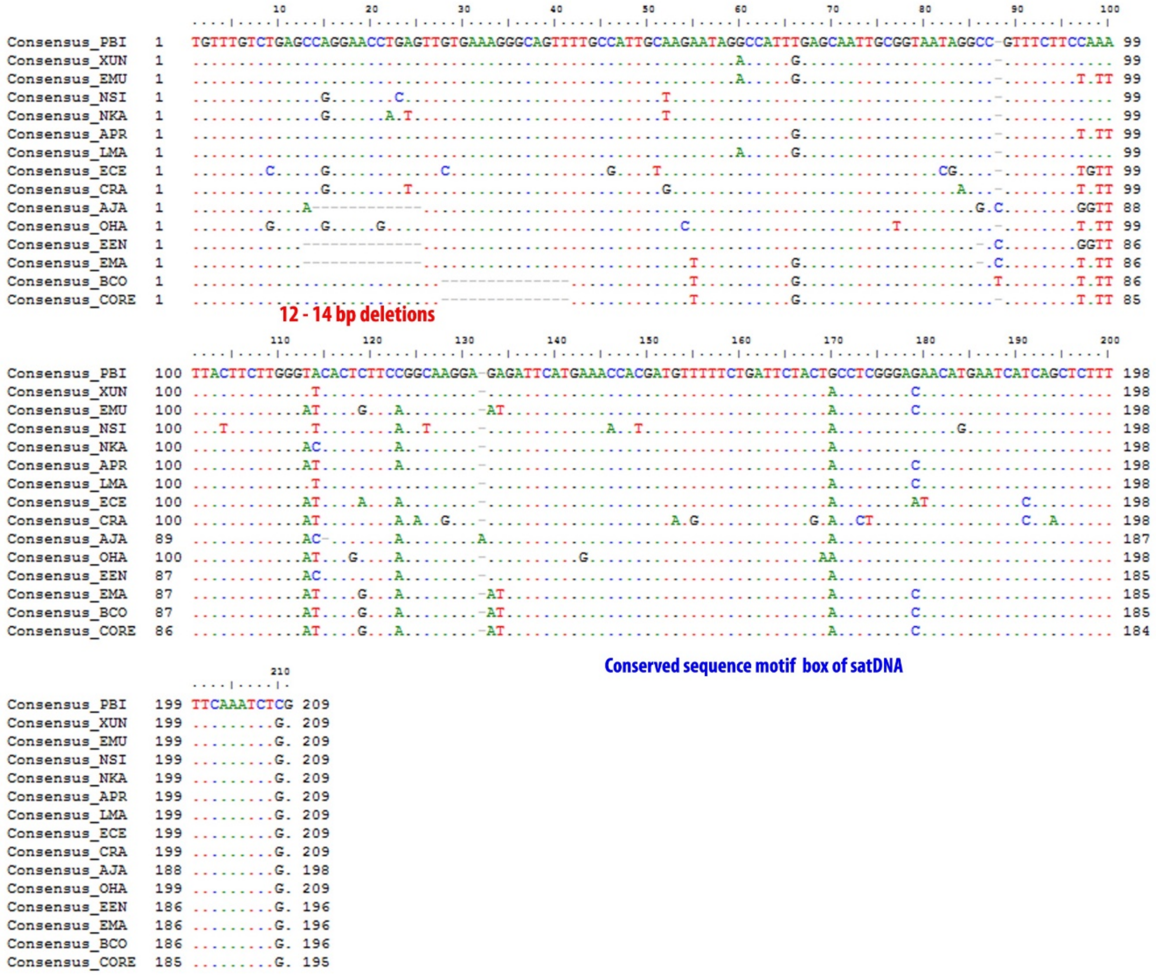

Supplementary Figure 3S (Thongchum et al.)

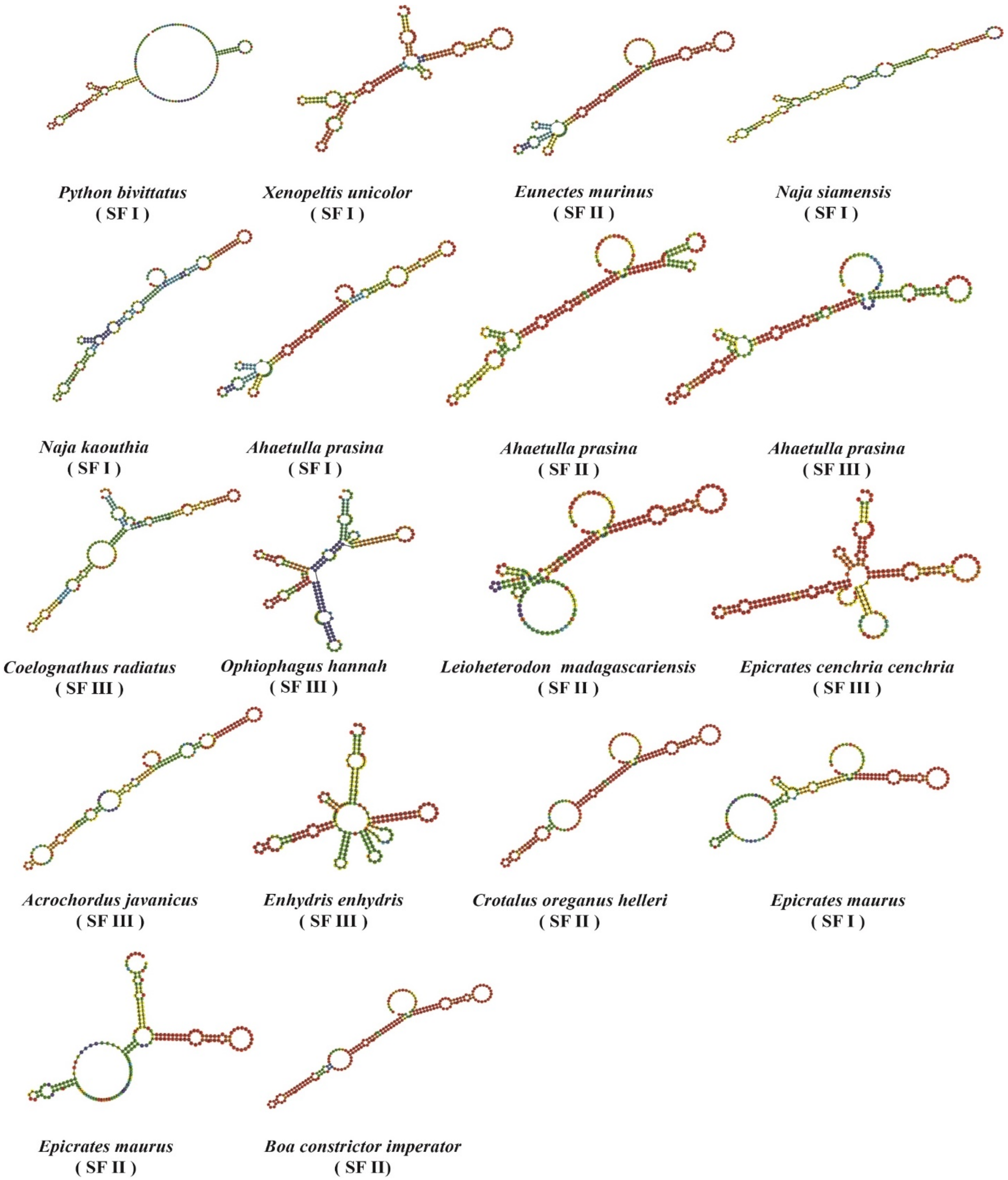

Supplementary Figure 4S (Thongchum et al.)

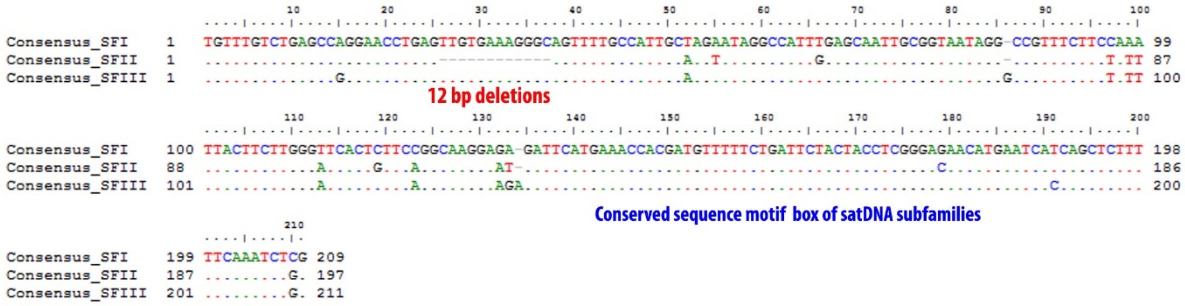

Supplementary Figure 5S (Thongchum et al.)

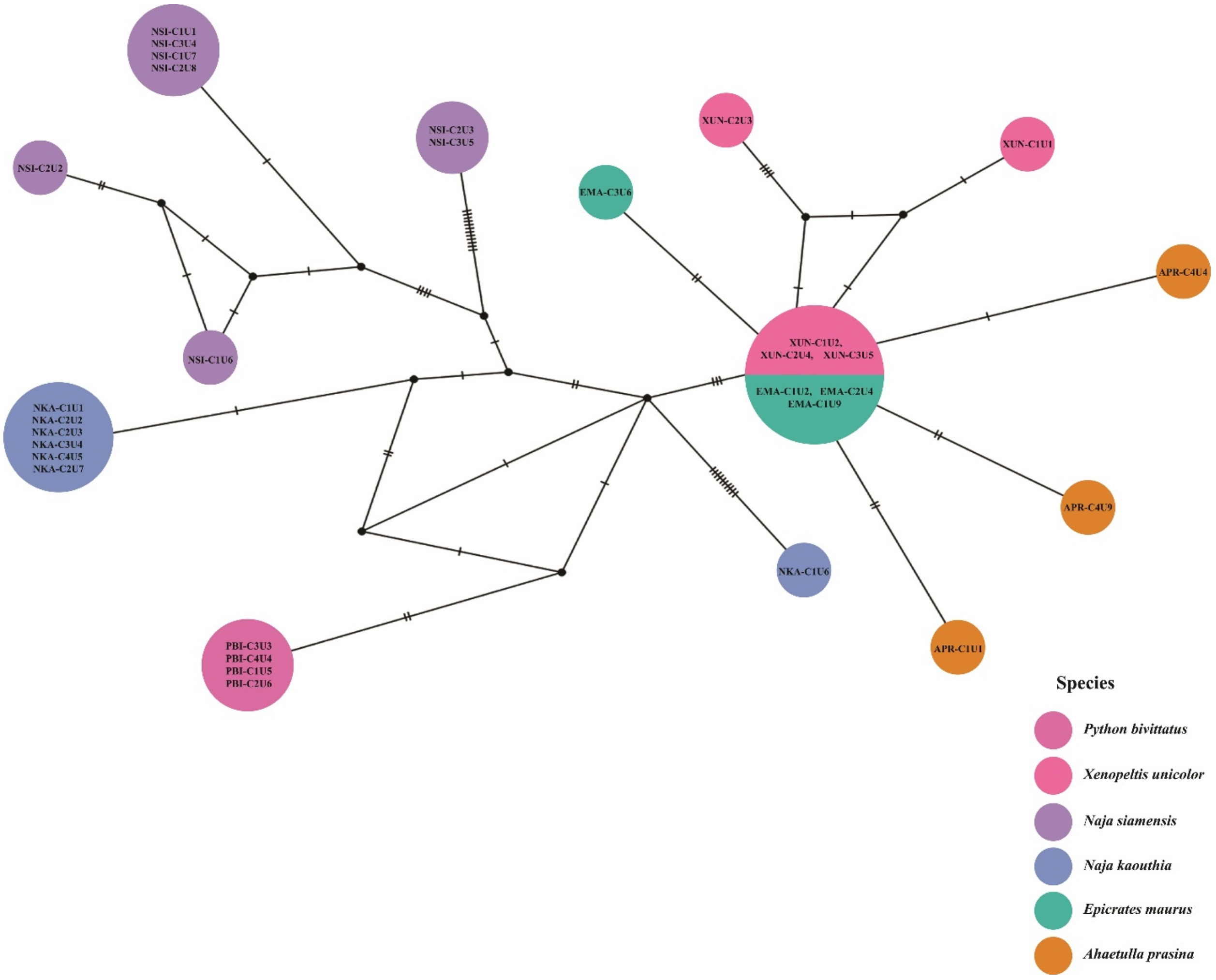

Supplementary Figure 6S (Thongchum et al.)

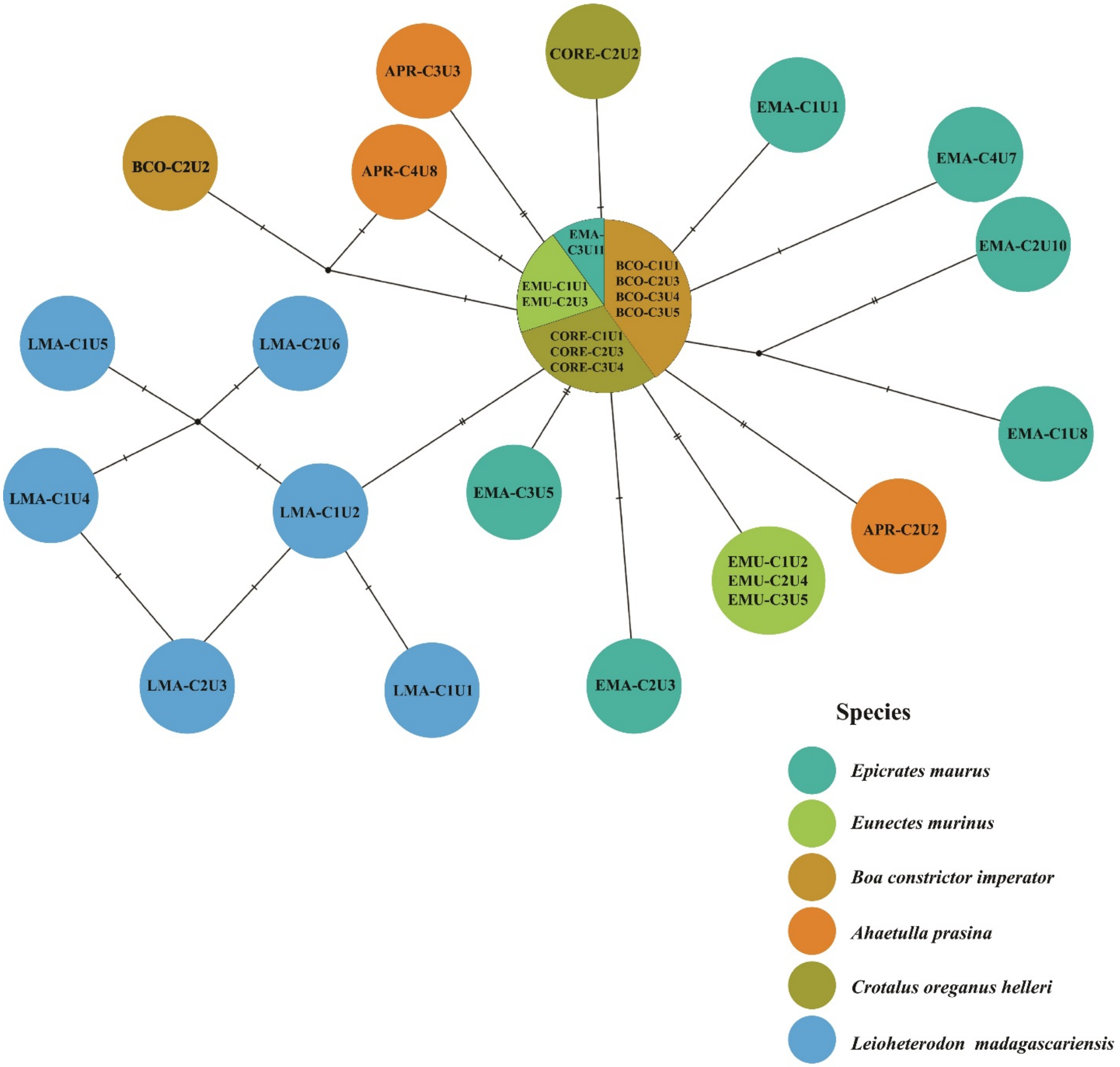

Supplementary Figure 7S (Thongchum et al.)

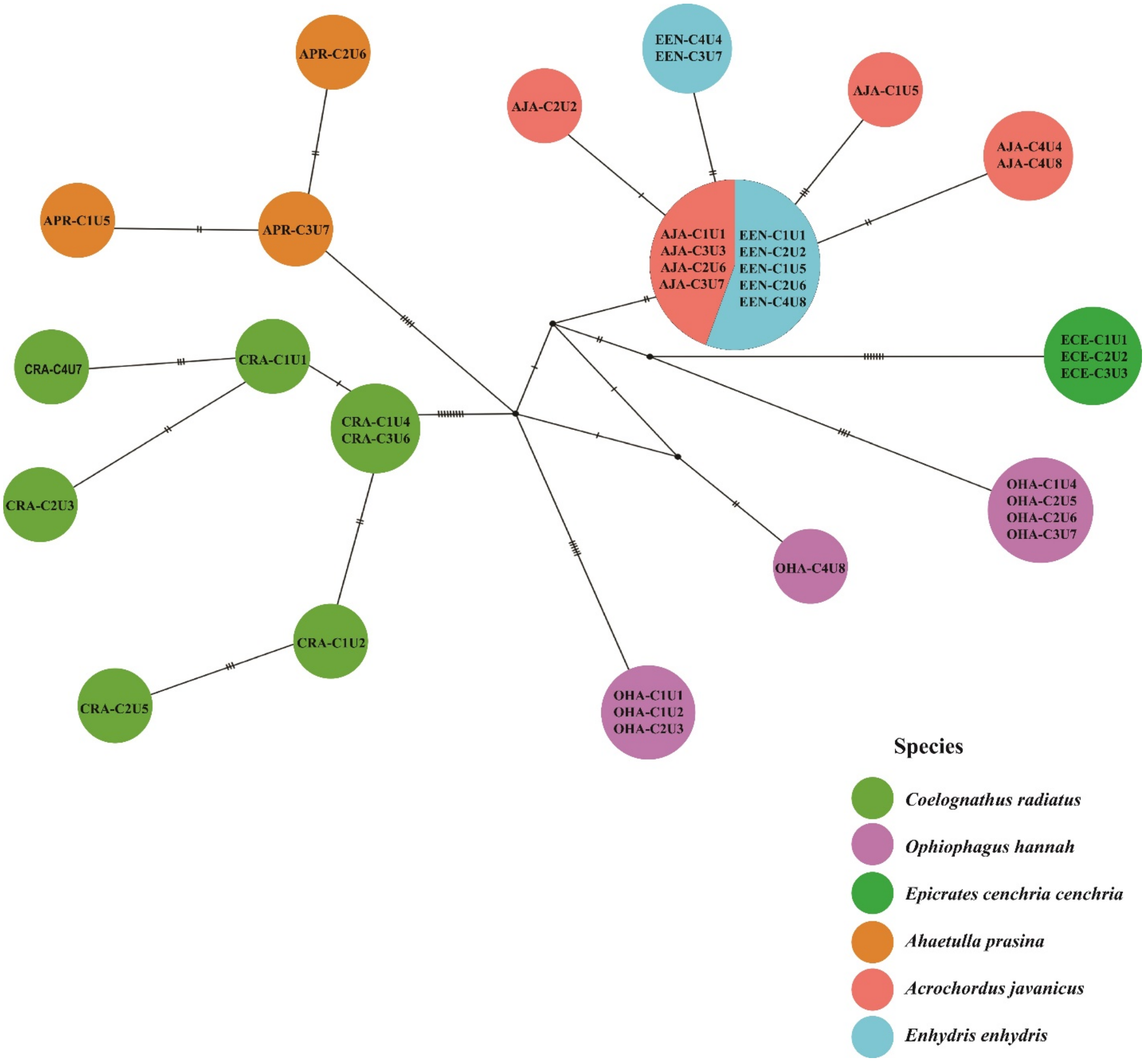

Supplementary Figure 8S (Thongchum et al.)

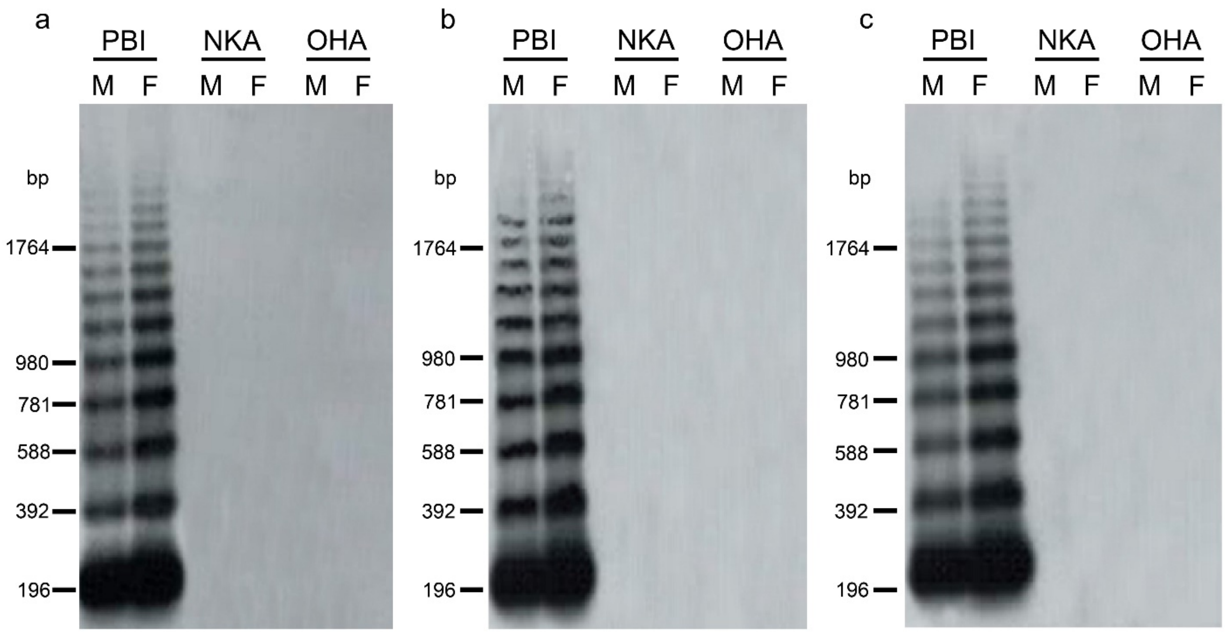

Supplementary Figure 9S (Thongchum et al.)

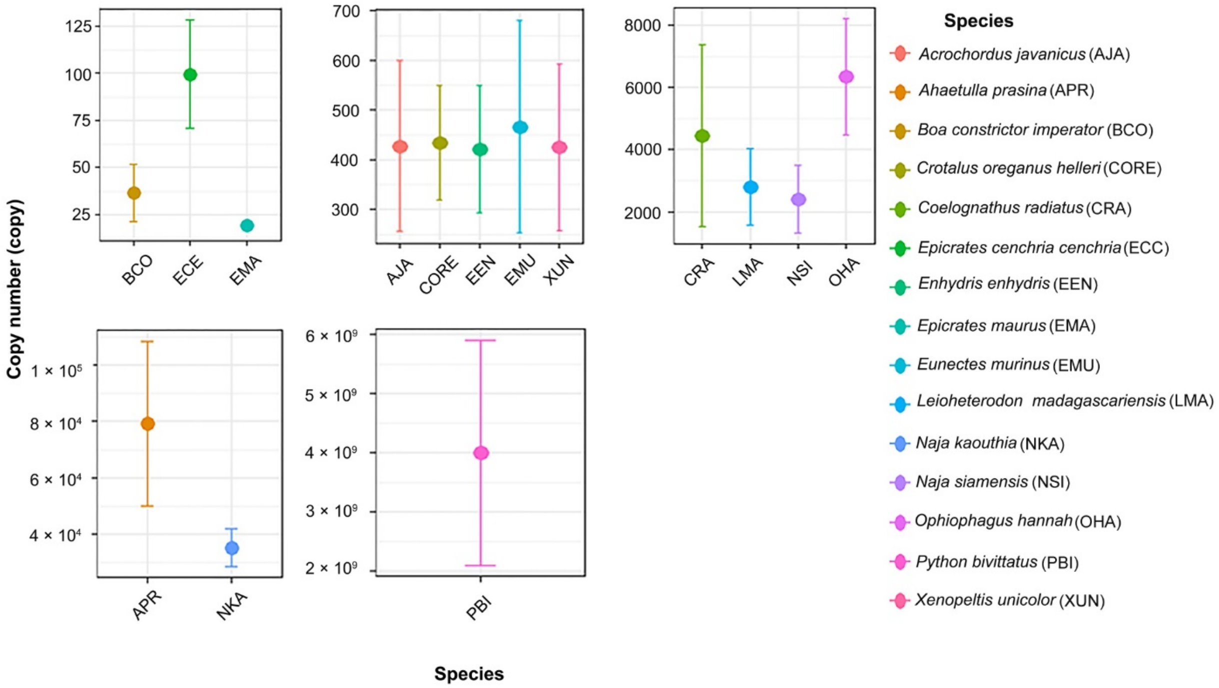

Supplementary Figure 10S (Thongchum et al.)

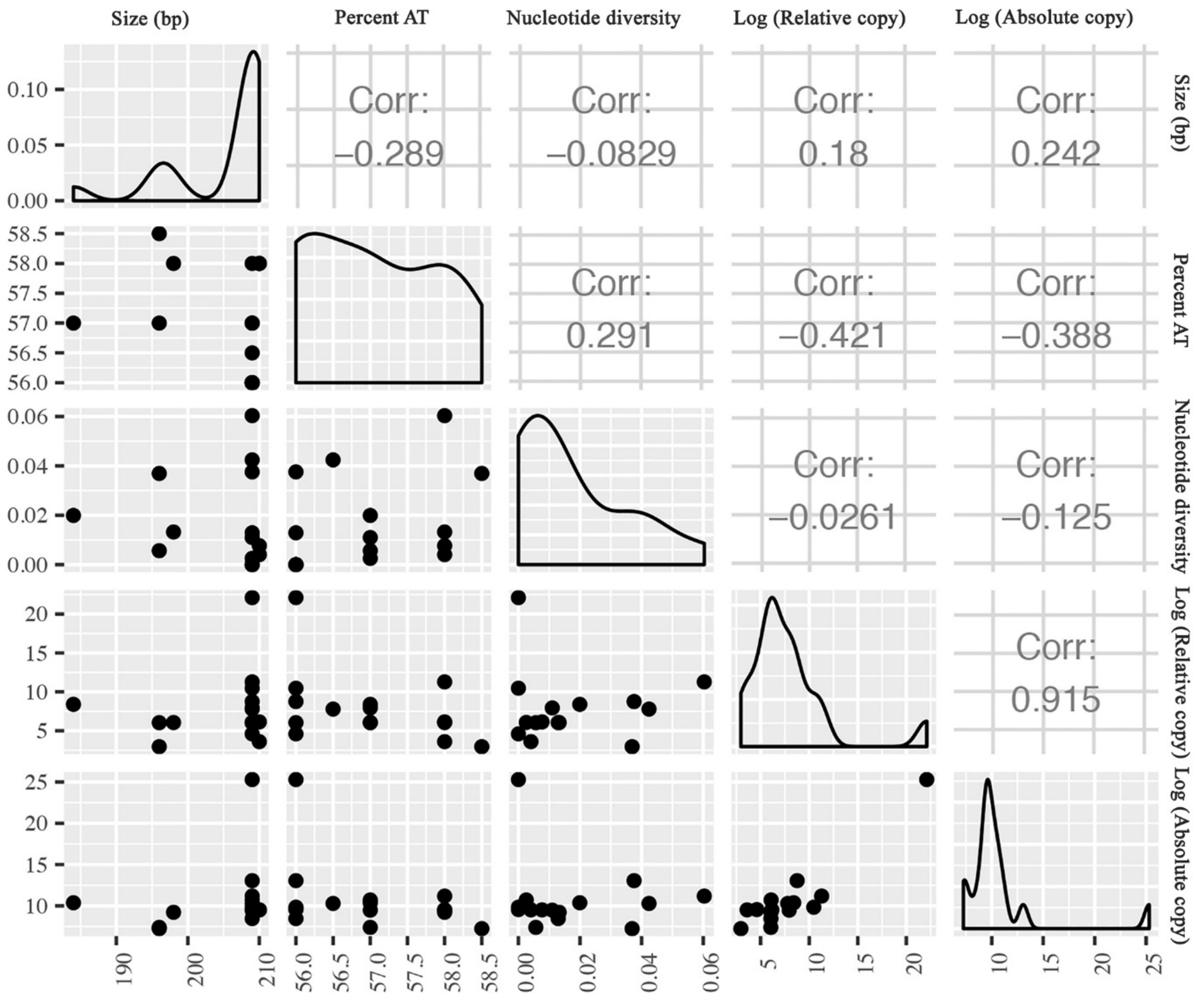

Supplementary Figure 11S (Thongchum et al.)

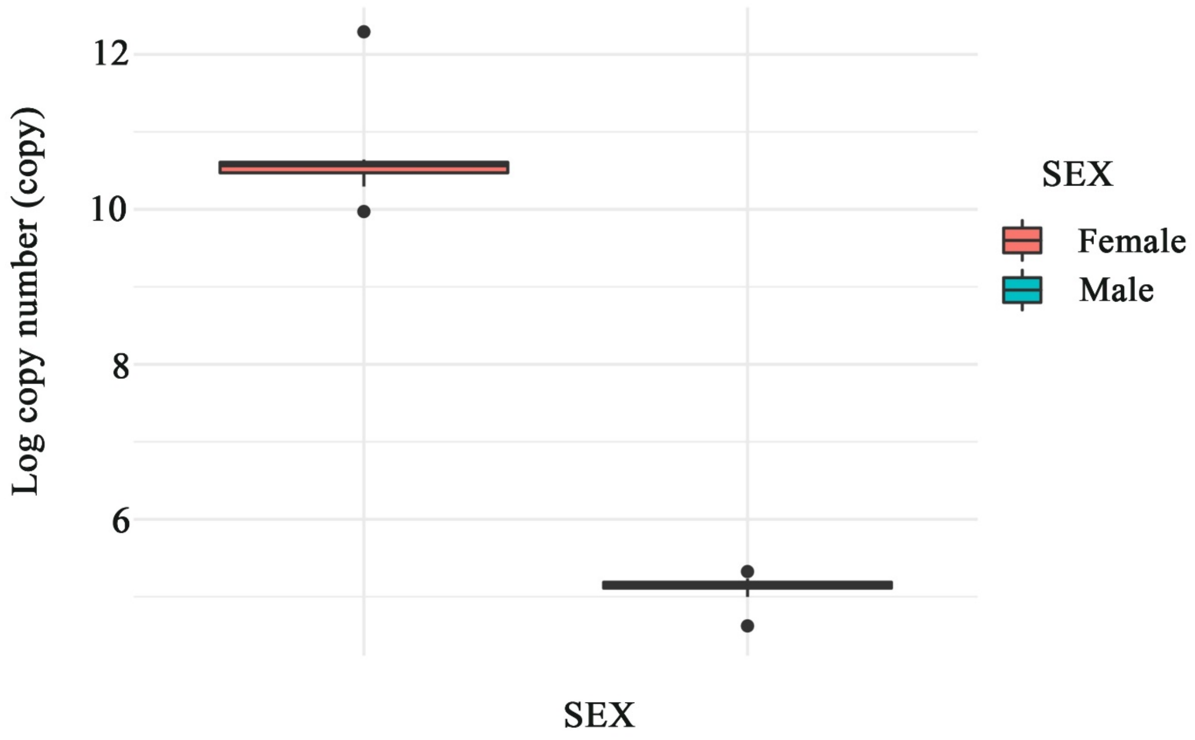

Supplementary Figure 12S (Thongchum et al.)

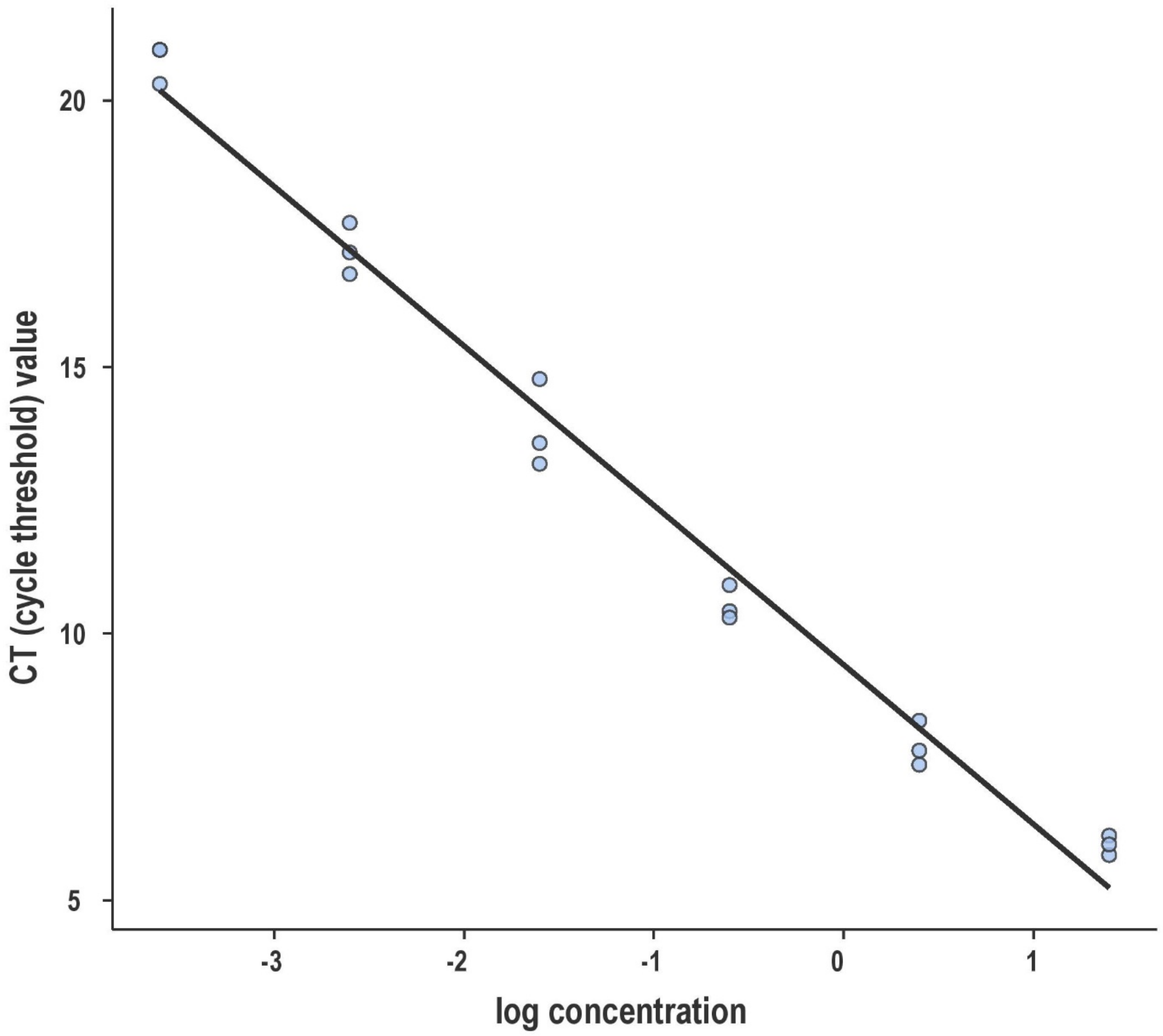

Supplementary Figure 13S (Thongchum et al.)

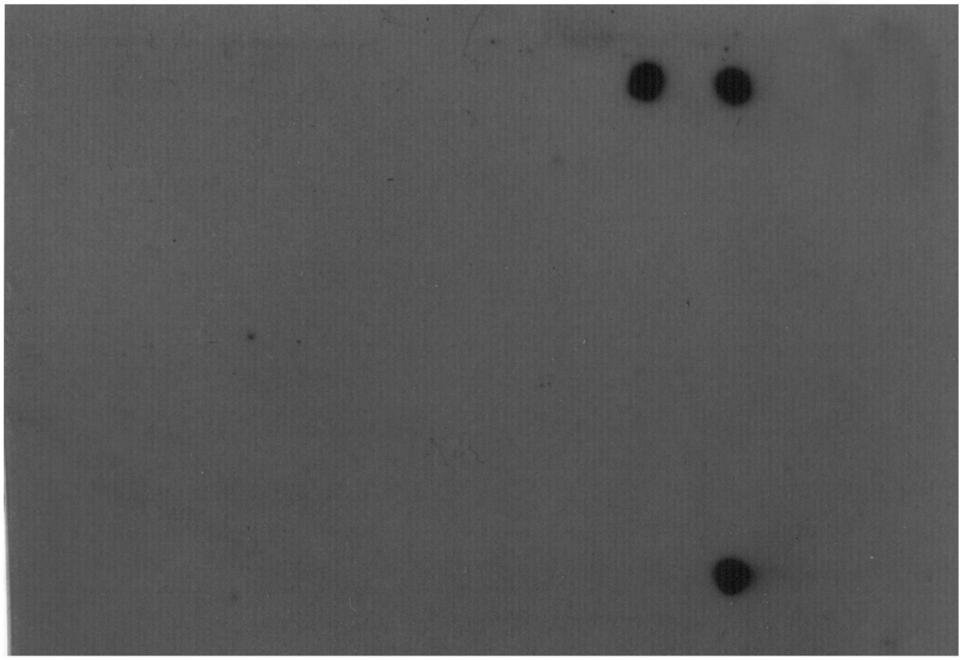

CRU-M	CRU-F	EMA-M	EMA-F	XUN-M	XUN-F	PBI-M	PBI-F	AJA-M	AJA-F
DSI-M	DSI-F	CCE-M	CCE-F	HBU-M	HBU-F	EEN-M	EEN-F	LMA-M	LMA-F
PGU-M	PGU-F	NKA-M	NKA-F	NSI-M	NSI-F	OHA-M	OHA-F	BCA-M	BCA-F
BFL-M	BFL-F	OFA-M	OFA-F	APR-M	APR-F	BDE-M	BDE-F	GOX-M	GOX-F
CFL-M	CFL-F	XRA-M	XRA-F	CRA-M	CRA-F	PMU-M	PMU-F	EMU-M	ECE-M
BCO-F	TAL-M	CORE-M	CDU-F	BGA-F	CHO-M	BCY-M	BCO-M	BCYA-M	RNI-M
ANA-F	CPA-F	COR-F	GPR-F				PBI-DdeI satDNA		

Dot blot hybridization (Original file for Supplementary Fig. 1S)

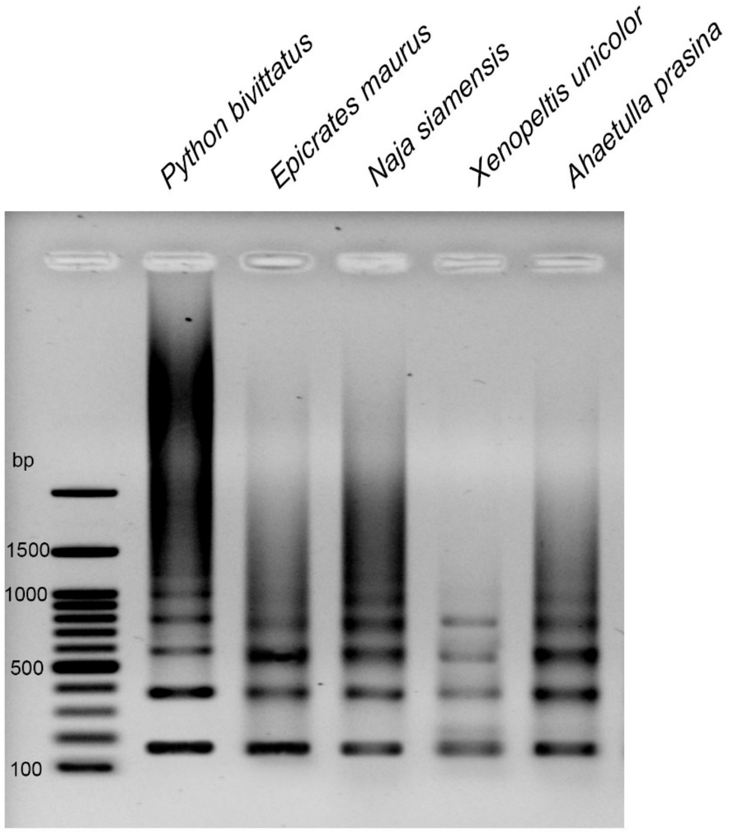

Agarose gel electrophoresis (Original file for Supplementary Fig. 2S)

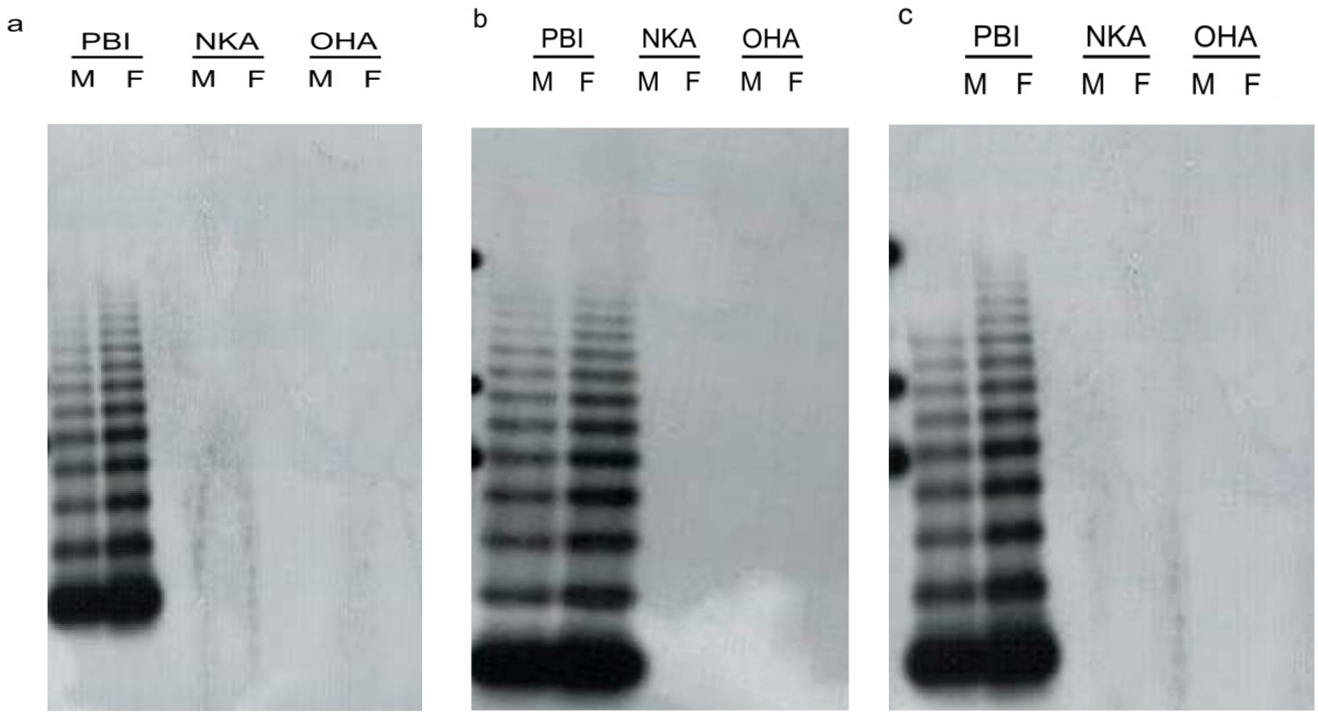

Southern blot hybridization (Original file for Supplementary 9S)
